# Genome-wide association analysis reveals variants on chromosome 19 that contribute to childhood risk of chronic otitis media with effusion

**DOI:** 10.1038/srep33240

**Published:** 2016-09-16

**Authors:** Elisabet Einarsdottir, Lena Hafrén, Eira Leinonen, Mahmood F. Bhutta, Erna Kentala, Juha Kere, Petri S. Mattila

**Affiliations:** 1 Folkhälsan Institute of Genetics, and Molecular Neurology Research Program, University of Helsinki, Helsinki, Finland; 2 Department of Biosciences and Nutrition, Karolinska Institutet, Huddinge, Sweden; 3 Department of Otorhinolaryngology, Helsinki University Hospital, Helsinki, Finland; 4 Children’s Surgical Centre, Phnom Penh, Cambodia

## Abstract

To identify genetic risk factors of childhood otitis media (OM), a genome-wide association study was performed on Finnish subjects, 829 affected children, and 2118 randomly selected controls. The most significant and validated finding was an association with an 80 kb region on chromosome 19. It includes the variants rs16974263 (*P* = 1.77 × 10^−7^, OR = 1.59), rs268662 (*P* = 1.564 × 10^−6^, OR = 1.54), and rs4150992 (*P* = 3.37 × 10^−6^, OR = 1.52), and harbors the genes *PLD3, SERTAD1, SERTAD3, HIPK4, PRX,* and *BLVRB*, all in strong linkage disequilibrium. In a sub-phenotype analysis of the 512 patients with chronic otitis media with effusion, one marker reached genome-wide significance (rs16974263, *P* = 2.92 × 10^−8^). The association to this locus was confirmed but with an association signal in the opposite direction, in a UK family cohort of 4860 subjects (rs16974263, *P* = 3.21 × 10^−4^, OR = 0.72; rs4150992, *P* = 1.62 × 10^−4^, OR = 0.71). Thus we hypothesize that this region is important for COME risk in both the Finnish and UK populations, although the precise risk variants or haplotype background remain unclear. Our study suggests that the identified region on chromosome 19 includes a novel and previously uncharacterized risk locus for OM.

Otitis media (OM) is a common childhood disease, and one of the most frequent reasons for doctor’s visits in childhood and for antibiotic therapy^1^. Surgical treatment of OM is also one of the most common reasons for children’s anesthesia^2^. Most children experience at least one episode of acute otitis media (AOM), but some develop recurrent acute otitis media (RAOM) or chronic otitis media with effusion (COME). The effusion in the middle ear accompanying OM is the most common reason for hearing impairment in children. There are a number of known risk factors for RAOM and COME, most importantly exposure to viral infections and a family history of OM. Male gender, lack of breastfeeding, extensive use of pacifiers, other atopic diseases, and parental smoking have also been identified as risk factors^3^.

Based on twin- and family studies, the heritability of OM has been estimated at 39–73%^4^,^5^,^6^,^7^ and a number of genetic studies have addressed the heritable component of OM. Several candidate gene studies as well as linkage studies, genome wide association (GWA) studies, and exome-sequencing have been carried out on OM.

Linkage studies, aiming to identify genetic regions associated with a phenotype in a family, have been performed on two family cohorts in OM. In the Minnesota family study of 371 affected individuals, linkage was found to COME and/or RAOM on chromosomes 10q26.3 (LOD 3.78) and 19q13.42-q13.43 (LOD 2.61)^8^. In the Pittsburg family cohort, linkage was discovered on 17q12 (LOD 2.85) and 10q22.3 (*P* = 2.6 × 10^−4^)^9^.

Most candidate gene studies on OM have focused on plausible risk genes involved in innate immunity or the function of cytokines^10^. The innate immune system is fully developed at birth and is especially important until the adaptive immunity system is mature. Among innate immunity genes, *MBL* (Mannose-binding lectin)^11^,^12^, *TLRs* (Toll-like receptors)^4^,^7^,^13^,^14^,^15^, *CD14* (Cluster of differentiation 14)^5^, surfactant, several interleukins (*IL6*^6^,^7^,^16^,^17^*, IL10*^7^,^16^,^18^,^19^*, IL1α*^20^*, IL1β*^19^,^21^), other cytokines, such as *TGFβ1* (Transforming growth factor beta 1)^18^,^22^*, IFNγ* (Interferon gamma)^9^*, TNFα* (Tumor necrosis factor alpha)^6^,^16^,^17^, and the cytokine receptor *CX3CR1* (Chemokine, CX3C motif, receptor 1)^21^, have been associated with OM, as well as the genes *FBXO11* (F-box only protein 1)^23^,^24^, *SMAD2* (SMAD family member 2)^14^,^23^, and *SMAD4*^14^,^23^ in the TGFβ1 pathway. Other candidate genes found to associate to OM include the mucins^14^,^15^,^25^, *SCN1B* (Sodium channel subunit beta-1)^15^, *SERPINE1* (serpin peptidase inhibitor, clade E (nexin, plasminogen activator inhibitor type 1), member 1, also known as *PAI1*)^7^, *SLC11A1* (Solute carrier family 11, Member 1)^25^, *CPT1A* (Carnitine palmitoyltransferase I, liver)^26^, and the HVRII region of mitochondrial DNA^26^.

GWA studies are commonly used in the hunt for genetic risk factors underlying complex diseases. In GWA studies, a great number of single nucleotide polymorphisms (SNP) are genotyped in large cohorts of cases and controls. Differences in marker allele frequencies between cases and controls indicate a possible locus of interest. The GWA method is relatively hypothesis-free, and P values <5 × 10^−8^ are generally considered genome-wide significant^27^. To date there are only two published GWA studies on OM. The first involved an Australian cohort of 416 affected individuals and 1075 controls^24^, where no genome-wide significance was reported, and the strongest findings were not replicated either in an Australian family cohort or in a North-American family cohort^18^. Allen *et al.* performed the second GWA study on OM in a North American family cohort on 143 families, with 373 affected probands. They did not obtain genome-wide significance in the initial study, but by analyzing the strongest findings in another North American family cohort of OM, they replicated the result for the variant rs10497394 in an intergenic region on chromosome 2 (*P*(meta-analysis) =1.52 × 10^−8^)^22^.

The first large-scale sequencing study on OM was performed by Santos-Cortez *et al.* By exome-sequencing two second cousins in a Filipino isolate where the prevalence of OM is almost 50%, a variant in *A2ML1* (α-macroblobulin-like 1) was identified as a novel rare, but high risk, candidate variant for OM^19^.

Taken together, OM is a complex genetic disease^28^, where both common, low risk genetic variants, as well as rare, high-risk variants may add up to the genetic risk. Several genetic studies have been conducted, with positive findings not always replicating in other cohorts. There is still a vast amount of knowledge lacking to fully explain the genetic risk component of OM.

A GWA study is an efficient way to identify association to common variants without an a priori hypothesis. We have recruited a large cohort of Finnish OM families where heritability has been previously estimated^17^. This well phenotyped cohort originates from a clinically and genetically homogeneous setting, making it well suited for a GWA study. Our aim was to identify genetic variants associated with OM to better understand the underlying pathophysiology of OM and potentially help optimize its treatment.

## Results

### Genotype Materials

After quality control, we had genotype information from 803 OM cases and 2073 controls, comprising 1485 males and 1391 females. The combined genotype dataset consisted of 319,683 genetic variants after all QC steps. The genotyping rate in this dataset was 99.9% and all individuals had a 95% call rate or higher. Quantile-quantile plots (QQ-plots) for all OM, COME, and RAOM are shown in Supplementary Fig. 1. The genomic inflation factor for all OM versus controls was 1.028, it was 1.021 for COME, and 1.029 for RAOM. There was thus no indication of any hidden stratification between cases and controls, resulting in systematic inflation of positive association signals. Imputation was performed on this dataset, but the results did not strengthen the findings of the study and signals seen in imputed data could not be validated (data not shown). The imputed data (available upon request) were thus not used in subsequent analyses.

### Association

**Figure 1:**
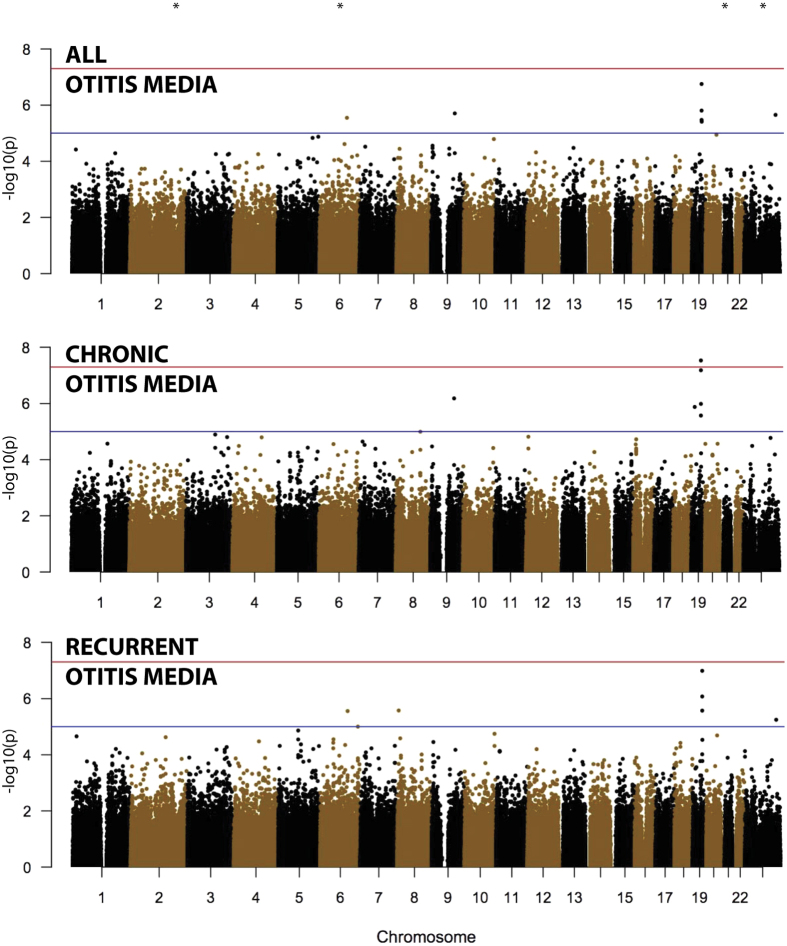
Manhattan plots of the association of OM (all patients), COME, or RAOM with each chromosome. The four very highly associated markers (on chromosomes 2, 6, 21 and X) are not shown, but their approximate position is marked by stars at the top of the figure. Each dot represents one genetic variant, the x-axis shows the approximate position of each marker on chromosomes 1 to X. The y-axis shows the −log10(p) value, with higher values indicating more significant association. The red line at −log10(p) =7.3 indicates the threshold for fully genome-wide significant association, the red line at 5 indicates suggestive association.

Table 1A shows the association of four highly associated markers on chromosomes 2, 6, 21 and X, as well as the association of markers on chromosome 19q. No other markers reached genome-wide significance or were deemed to be plausible true association signals. Figure 1 shows Manhattan plots of the association of OM (all patients), COME, and RAOM. The four very highly associated markers are not shown, but their approximate position is marked by stars at the top of the figure. The association to these four markers was not supported by surrounding markers, and is thus not likely to represent true association signals. The association to these four markers, and the lack of association of markers in LD with them, is illustrated in Supplementary Fig. 2.

**Figure 2:**
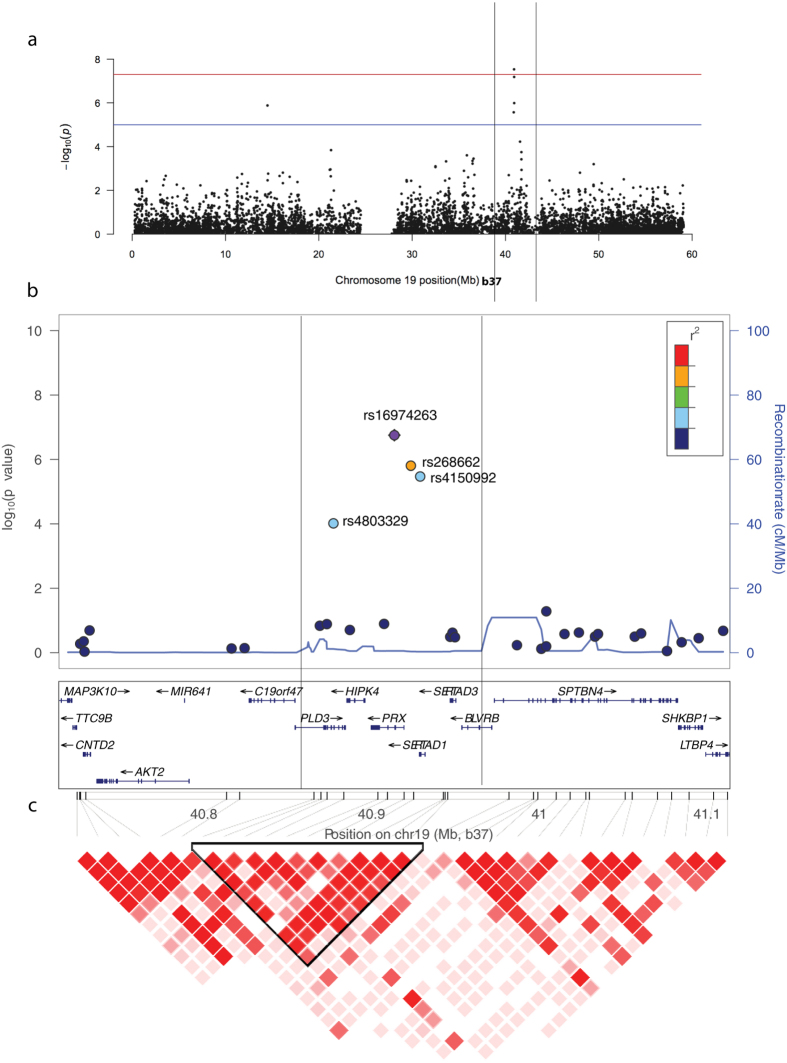
(**a**) GWA study Manhattan plot for chromosome 19. (**b**) Association to OM (all affected) in markers within a 200 kb region surrounding rs16974263 and their estimated LD with each other. The genes that are located within the region are shown, as well as recombination patterns (plotted based on data from the hg19/1000 Genomes dataset, Nov 2014 EUR population). (**c**) Pairwise D’ LD between all markers in the region based on our data. The selected markers constitute a block of strong LD, containing our associated markers.

**Figure 3:**
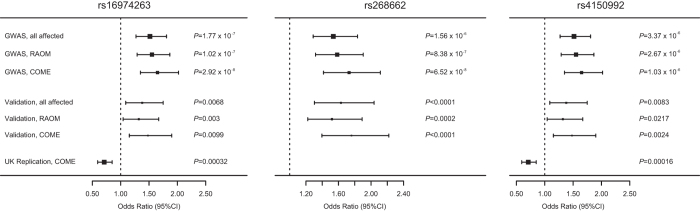
Forest plot data for the three highly significant genetic variants on chromosome 19. Each plot shows the odds ratio (OR) for each dataset as a black box, and the horizontal lines indicate the 95% CI for each OR.

Marker rs16974263 showed the strongest association to OM (all patients, COME or RAOM) in the horizontal LD segment on chromosome 19q (Fig. 2, Fig. 3, Supplementary Table 1, Supplementary Fig. 3). Allele A of rs16974263 was found to have a frequency of 14.1% when looking at all cases, and 9.4% in controls (*P* = 1.76 × 10^−7^, OR = 1.59 (1.33–1.89)). This association remained significant (*P* = 0.011) even after Benjamini-Hochberg (FDR_BH) false-discovery rate correction, as applied through PLINK (Supplementary Table 2). Allele C of rs268662 had a frequency of 13.7% in all cases, and 9.4% in controls (*P* = 1.56 × 10^−6^, OR = 1.53 (1.29–1.83)). Rs4150992 allele G was found at a frequency of 13.6% in all cases, and 9.4% in controls (*P* = 3.37 × 10^−6^, OR = 1.52 (1.27–1.81)). Marker rs4803329 showed weaker association, allele A had a frequency of 12.2% in all cases, and 8.8% in controls (*P* = 9.63 × 10^−5^, OR = 1.44 (1.20–1.73)).

Comparing only COME patients to controls, we saw association to the same four markers on chromosome 19, with slightly stronger association even with fewer cases (Fig. 2, Fig. 3, Supplementary Table 1, Supplementary Fig. 3). Allele A of rs16974263 was found to have a frequency of 15.3% when looking at COME cases, and 9.4% in controls (*P* = 2.92 × 10^−8^, OR = 1.75 (1.43–2.14)). Allele C of rs268662 had a frequency of 15.2% in COME cases, and 9.4% in controls (*P* = 6.52 × 10^−8^, OR = 1.73 (1.42–2.12)). Rs4150992 allele G was found at 14.7% frequency in COME cases, and 9.4% in controls (*P* = 1.03 × 10^−6^, OR = 1.65 (1.35–2.02)). The marker rs4803329 showed weaker association, with allele A found at a frequency of 13.7% in COME cases and 8.8% in controls (*P* = 2.68 × 10^−6^, OR = 1.64 (1.33–2.02)).

The association of COME to marker rs16974263 was genome-wide significant (*P* < 5 × 10^−8^) and the association to rs16974263 and rs268662 was significant even after a Bonferroni correction for 319,683 markers (*P* = 0.0093 and *P* = 0.02, respectively) (Supplementary Table 2). As the majority of patients with COME also have RAOM, we considered performing association analysis also on the patients suffering only from COME (N = 78) to see if the association was more strongly driven by the COME phenotype. This number of cases was, however, too low for meaningful analyses and was not followed up further (data not shown).

Comparing only RAOM patients to controls, we saw a very similar pattern of association to the same four markers on chromosome 19 (Fig. 2, Fig. 3, Supplementary Table 1, Supplementary Fig. 3). Rs16974263 allele A was found at a frequency of 14.5% in RAOM cases and 9.4% in controls (*P* = 1.02 × 10^−7^, OR = 1.64 (1.36–1.96)). Allele C of rs268662 had a frequency of 14.1% in RAOM cases, and 9.4% in controls (*P* = 8.38 × 10^−7^, OR = 1.58 (1.32–1.91)). Rs4150992 allele G was found in 13.9% of RAOM cases, and 9.4% of controls (*P* = 2.67 × 10^−6^, OR = 1.55 (1.29–1.87)). Allele A of rs4803329 had a frequency of 12.4% in RAOM cases, and 8.8% in controls (*P* = 9.59 × 10^−5^, OR = 1.47 (1.21–1.78)). The association of rs16974263 was still significant after Bonferroni correction (*P* = 0.033), and the association of both rs16974263 and rs268662 remained after FDR_BH correction (*P* = 0.0065 and *P* = 0.044, respectively) (Supplementary Table 2). Adding sex as a covariate in the GWA analysis yielded very similar association results (data not shown) and was not followed up further.

Two-, three- or four-marker haplotypes of the four associated markers, in either all OM, RAOM or COME, did not yield stronger association than the signal contributed by rs16974263 alone (data not shown).

### Validation

As our case and control genotype data originated from two different sources, it was crucial to verify our strongest association signals from the GWA study by another method and to genotype cases and controls together. All genotyped markers were in HWE (*P* > 0.05) in controls. Association to three of the four very highly associated markers, rs3821170 (on chr2), rs2406176 (on chr21) and rs4825724 (on chrX), was not replicated. Assay design for rs885932 (on chr6) failed (Table 1B). Furthermore, the concordance of the genotypes in the GWA study versus the follow-up genotyping was lower in these markers. This indicates that the association to these markers in the initial GWA study was likely to be an artefact, caused by unknown genotyping errors or differences in assay design.

Of the four markers on chromosome 19 showing association to OM, rs16974263, rs4150992 and rs268662 were successfully genotyped in the validation dataset. Association of these markers with OM susceptibility was confirmed in the validation dataset (Table 1B), with similar allele frequencies and odds ratios (OR) as in the initial GWA study. This indicated that the chromosome 19 findings constitute a plausible true association.

### Replication

In order to assess the reproducibility of our findings in an independent dataset and population, we genotyped two of the validated associated markers on chromosome 19 in an independent cohort of UK families affected by COME. This dataset consisted of 1507 nuclear families and 1247 successfully genotyped full trios. Both markers had call rates >95%. TDT analysis revealed association of both rs16974263 and rs4150992 (Table 1C) with COME. Rs16974263 allele A was transmitted 196 times, un-transmitted 274 times (*P* = 0.00032, OR = 0.71 (0.60–0.86)), rs4150992 allele G was transmitted 206 times, un-transmitted 290 times (*P* = 0.00016, OR = 0.71 (0.59–0.85)). The odds ratios for this association were similar to the ones found in the Finnish population, but to the opposite alleles. The UK dataset was assumed to be approx. 94% Caucasian. Analysis using only individuals with known Caucasian origin (proband N = 636) yielded highly similar results as using the whole dataset (data not shown).

### *In Silico* Analysis On Chromosome 19

Figure 2 shows a more detailed view of the association to chromosome 19q. The association signal most likely constituted a single association signal tagged by four markers within a single LD block of approx. 80 kb at 40,869 kb to 40,950 kb on chromosome 19. It is likely that whatever is driving the association to OM susceptibility lies within this region.

Our OM susceptibility candidate region contains the genes *PLD3* (Phospholipase d family, member 3), *HIPK4* (Homeodomain-interacting protein kinase 4), *PRX* (Periaxin), *SERTAD1* (Serta domain-containing 1), *SERTAD3* (Serta domain-containing 3), and *BLVRB* (Biliverdin reductase b). The first exon and promoter regions of *PLD3* and *BLVRB* were not within the immediate region of interest, but genetic variation in other parts of either gene cannot be excluded.

We surveyed data from the FANTOM5 consortium to look for transcription start sites in the LD block containing our association signal and to see where the transcripts from each start site might be expressed. We saw that the associated region contained two very strong transcription start sites, one within the *SERTAD1* gene and one at the start of the *SERTAD3* gene. It also showed a strong transcription start site in the middle of the *PLD3* gene. The expression levels of other genes appeared considerably lower in all of the >200 tissues.

We looked for ENCODE functional elements in the region covering the six genes. The region contained eleven likely CpG islands, seven probable strong expression start sites, dozens of likely transcription factor binding sites, and conserved regions overlapping with these.

Supplementary Table 3 shows the scores and P values for each of the six candidate genes in the ToppGene multi-source data analysis within the chromosome 19 associated region. *SERTAD3* and *HIPK4* appear to be less likely candidates for involvement in OM (P values 0.32 and 0.72, respectively), whereas *PLD3*, *BLRVB*, *SERTAD1* and *PRX* are more similar to previously described OM genes.

We used WebGestalt to look at the gene ontology categories of the previously known OM genes, to learn into which functional categories each gene might cluster, as well as seeing where our six candidate genes from chromosome 19 might end up in relation to them. Supplementary Fig. 4 shows the results of this analysis. For suggested OM associated genes, we observed a strong enrichment of genes involved in Positive regulation of transport (*P* = 1.68 × 10^−13^) and Regulation of cytokine production (*P* = 1.65 × 10^−12^). There was also a highly significant enrichment of genes involved in response to bacteria and lipopolysaccharide (e.g. the category “Response to lipopolysaccharide”, *P* = 1.69 × 10^−13^). We found enrichment of genes involved in e.g. cytokine activity, cytokine receptor binding, as well as pattern recognition receptor activity (*P* = 4.04 × 10^−5^). We also saw strong enrichment of genes in the extracellular region and matrix (eg. the category “Extracellular region part”, *P* = 1.09 × 10^−9^). None of our genes were included in any of the categories that were significantly enriched in known OM genes. This might be due to lack of information about these genes or the identification of a novel mechanism for OM risk. Supplementary Table 4 shows a summary of known OM risk variants and how they compare to the association in the current study.

## Discussion

We report a genome-wide significant association of OM to an approximately 80 kb region on chromosome 19 based on a Finnish cohort of 803 successfully genotyped affected children and 2073 controls. This association was validated on a different genotyping platform, and association to the same locus was also found in a UK population (albeit with opposite risk effects). In the analysis of 512 patients with COME, the association reached genome-wide significance. This novel OM risk locus contains the genes *PLD3, HIPK4, PRX, SERTAD1, SERTAD3,* and *BLVRB.*

In the replication analysis in the UK population of mainly COME patients, the significant results were to the opposite marker alleles. Getting significant association of the same trait to the opposite alleles of the same genetic markers is counterintuitive, but such findings in other studies have been found previously. Examples include PepT1 oligopeptide transporter (*SLC15A1*) gene polymorphisms in inflammatory bowel disease in Finnish vs. Swedish populations^29^ as well as in *CD14* and *CC16* variants and allergy in Finnish vs. Russian Karelians^30^. Our results concerning the opposite marker alleles might be due to a number of factors. Firstly, it is possible that the genetic background underlying OM in the two populations may be distinct, despite the same genes being important. Secondly, differences in the environment of the two populations, e.g. differences in respiratory tract pathogen load and in exposure to antigens causing hypersensitivity reactions may elicit different pathogenic pathways. In this context it is intriguing that in a systems biology study of LPS stimulation of dendritic cell subpopulations, two of the genes within the region on chromosome 19 of interest, namely *PLD3* and *SERTAD1,* have been found to be selectively regulated in CD11b and CD8 dendritic cell subpopulations, respectively, which are distinct dendritic cell populations that regulate Th2 and Th1 immune responses, respectively^31^. Thus, one might argue that the apparent contradiction of opposite risk alleles might be due to differences in pathogenic pathways caused by different environmental factors in Finnish vs. British children although the ultimate result is similar in terms of prolonged middle ear effusion characteristic of COME.

In a previous linkage study on OM there was an associated region on chromosome 19 (q13.42-q13.43)^8^,^32^. That region does not overlap with the Chromosome 19q13.2 region presented in the current study. Previously suggested OM candidate genes on chromosome 19, *TGFβ* (19q13.2)^18^,^22^ and *SCN1B* (19q13.12)^15^, also do not overlap with the novel region reported here. An earlier GWA study on OM found significant association to the marker rs10497394 (2q31.1) on the chromosome 2^22^. This marker was genotyped in our data, but did not associate to OM. Supplementary Table 4 shows a comparison of previous reports of association to OM and our current GWA data.

Several of the genes within the 80 kb segment on chromosome 19 could potentially influence the pathogenesis of COME. We looked further at their expression levels in FANTOM5, the GTEx Portal, and the Human Protein Atlas. According to FANTOM5, ***PLD3***, Phospholipase d family, member 3, is transcribed from two distinct promoters and transcription from the second promoter is strongly activated by LPS in monocyte-derived macrophages. Rare coding variants of *PLD3* have been associated with the risk of late onset Alzheimer’s disease^33^. ***SERTAD1**,* Serta domain-containing 1, has according to FANTOM5 one main promoter and transcription from it is strongly up-regulated in monocyte-derived macrophages upon response to influenza virus and in CD14 + monocytes upon response to BCG *Mycobacterium* bacteria. It is of interest that both influenza virus and mycobacteria can elicit Th1 immune responses^34^,^35^. ***SERTAD3*** is homologous to *SERTAD1* and is up-regulated in MCF7 breast cancer cell lines (FANTOM5). ***HIPK4***, homeodomain interacting protein kinase 4, belongs to the serine threonine protein kinase family and seems, according to FANTOM5 and the GTEx Portal, to be exclusively expressed in testis. ***PRX***, periaxin, has two main transcription start sites and is mainly expressed eye, spinal cord and other neuronal tissues, as well as in lung, mesenchymal stem cells, and muscle. *PRX* is expressed in the tibial nerve and in lung, according to the GTEx Portal, whereas PRX protein expression is found in kidney and in peripheral nerves. ***BLVRB***, biliverdin reductase B, is according to FANTOM5 expressed in CD34 + cells, as well as in whole blood and in monocyte-derived macrophages responding to certain influenza infections and in bronchial epithelial cells. According to the GTEx portal and Human Protein Atlas *BLVRB* RNA and BLVRB protein are expressed and found in almost all tissues. Two of the genes in this region, namely *PLD3* and *SERTAD1,* are particularly interesting as their expression is up-regulated by respiratory pathogens. Of note is that the 80 kB segment on chromosome 19 overlaps a region that is amplified in high-grade serous ovarian cancer, a cancer that is thought to originate from the peritoneal mucosa, and that this amplification predicts poor cancer survival^36^. Thus, a putative oncogene function of a gene in this region might contribute to mucosal hyperplasia and chronic inflammation characteristic of COME.

The ToppGene analysis suggests that *PLD3*, *BLVRB* or *SERTAD1* might be more likely OM candidate genes than *SERTAD3*, *PRX* or *HIPK4*. It is still, however, up for debate which gene should be considered the most plausible candidate gene, as this prioritization may differ considerably based on the presumed disease mechanisms or the queried datasets. None of our genes are included in gene categories that are enriched in previously suggested genes associated with OM, implying that a gene in this region may contribute to OM pathogenesis through a novel mechanism. Nevertheless, it is quite plausible that a gene, like *PLD3* or *SERTAD1* or both, that is highly up-regulated upon stimulation by pathogens and that is specifically regulated in dendritic cell subsets that control distinct immune response types^31^ would be a relevant candidate gene for OM.

Our study is based on a robust and large set of well-characterized cases that we could further stratify into disease sub-phenotypes. While our approach of matching our own case genotypes to control data of unselected Finnish individuals has some drawbacks, most of these are dwarfed by the strength our study gains from having more than 2000 population-based controls. This gave us power to potentially identify even weaker genetic effects and allowed us to gain full genome-wide significant association to chromosome 19 in a comparison of COME patients and controls. Our stringent initial quality control and the validation of associated markers contributed to robust association data.

We have previously found in a candidate gene study that the gene coding for the LPS receptor TLR4 is associated with the risk of especially early onset ROAM^20^. This signal was also detected in the present study but it did not reach genome-wide significance in the analysis of exhaustingly many distinct genetic polymorphisms. In the present report we that COME is associated with a gene region in chromosome 19 and that, although not completely resolved, polymorphisms in this region may possibly dissect COME into two putative entities with different pathogenic pathways. The strength of genetic studies of childhood OM is that they may help to dissect childhood otitis media into various entities arising by various pathogenic mechanisms. Thereby, genetic studies may help to understand the apparent heterogeneity of childhood otitis media that may be difficult to accomplish using other methods.

OM is poorly understood despite being such a clinically important disease. A full GWA study allows for a hypothesis-free study that did indeed reveal OM associated genes that would never have been hypothesized or tested as part of a targeted candidate gene study. We confirmed the association to the chromosome 19 locus in an independent dataset, although it remains unclear what are the mechanisms that led to the apparent contradiction of replication to the opposite alleles in the two different populations. This genomic region on chromosome 19 is thus clearly a confirmed OM susceptibility region and should be studied further. Our study has highlighted potentially novel mechanisms affecting the genetic risk to OM. Future studies will be needed to fully understand the factors driving the association to this region.

## Methods

### Study Subjects

The Finnish cases were combined from two cohorts (Table 2). The larger cohort consisted of 624 children from separate families, all suffering from RAOM or COME, recruited for genetic studies in OM. The second cohort comprised 205 children prospectively recruited to a randomized trial of adjuvant adenoidectomy for children having tympanostomy tubes for RAOM and/or COME. Study protocols were approved by the Ethics Committee at the Helsinki University Hospital (HUH) and the methods carried out in accordance with the approved study protocols. Written consent was obtained from all adult subjects or the children’s guardians.

The participants for the Finnish cohorts were recruited from patients referred to the Department of Otorhinolaryngology – Head and Neck Surgery at HUH, with a history of RAOM or COME. The criteria for RAOM were >3 acute otitis media (AOM) episodes in 6 months or >4 in 12 months^37^. The criterion for COME was effusion in the middle ear for more than 2 months, or effusion in the middle ear confirmed at tympanostomy tube insertion. Subjects were considered affected if they had RAOM or COME, or a history of insertion of tympanostomy tubes, used to surgically treat RAOM or COME.

We obtained informed consent from the children’s guardians, and information about the study subjects’ OM and medical history (as described previously, in^33^). DNA was extracted from peripheral blood using the FlexiGene DNA Kit (Qiagen, Hilden, Germany). The DNA from three patients was of low quality and thus not genotyped in the initial GWA study.

The control group for the GWA study comprised 2118 patients from the Finnish Health 2000 Survey, previously genotyped on the Illumina Infinium HD Human610-Quad BeadChip (Illumina, San Diego, CA, USA). These patients were adults (age > 30) selected to reflect the distribution of the Finnish population (http://urn.fi/URN:NBN:fi-fe201204193320^38^). As only *in silico* data was available for the first control group, we utilized an independent set of healthy Finnish blood donors (N = 778) as controls in the replication studies for the Finnish cohorts, as described in our previous candidate gene study^11^. Written informed consent was obtained from all control individuals.

The UK cohort was recruited from UK patients undergoing tympanostomy tube insertion together with their family members during April 2009 to November 2013. COME (middle ear effusion ≥3 months) was confirmed by effusion at myringotomy and RAOM was assessed by clinical history using the same criteria as in the Finnish index cohort. A study subject was considered affected if the criteria for COME and/or RAOM was fulfilled. A set of 1269 UK trios (parents and a child/children with OM), were included in the current study^11^. DNA was extracted from saliva samples collected by Oragene OG-250 (DNA Genotek Inc., Kanata, Ontario, Canada) using an automated system (LGC Genomics, Hoddesdon, UK). Approval for the study was granted by the NHS Oxfordshire Research Ethics Committee (study reference 08/H0605/109) and it was performed in accordance with relevant guidelines and regulations. Written informed consent was obtained from the children’s guardians.

### Genotyped datasets

1 ug of DNA from 826 cases was genotyped on the Illumina HumanOmniExpressExome array-8v1-2_A at the SNP&SEQ Technology Platform in Uppsala, Sweden according to standard protocols. Genotyping was performed using the Illumina Infinium assay and results on 964,193 SNP markers were analyzed using GenomeStudio 2001.1 from Illumina.

Genotypes were filtered to remove markers with <95% call rates and samples with call rates <99%. Markers showing discordance in duplicate samples were removed, as well as markers showing control genotypes inconsistent with previous genotype results (quality control as per setup at the SNP&SEQ Technology Platform).

The genotype data from both datasets were combined, retaining only markers found in both datasets. Markers with a minor allele frequency lower than 0.05 in either dataset were removed as minimally informative. Autosomal markers with Hardy-Weinberg equilibrium (HWE) P values <0.0001 in controls were also removed. X-chromosome markers were not filtered for HWE.

To minimize risk of allele-swap and differences in allele coding in the two datasets, leading to false-positive association, we removed any markers with wildly different allele frequencies (eg. 0% versus 100% of the same allele) in cases and controls. Imputation was performed using IMPUTE2^39^ and the 1000 Genomes v3 ALL reference dataset. PLINK v.1.09 (http://pngu.mgh.harvard.edu/~purcell/plink/40) was used to scan for more subtle incorrect strand assignments using the –flip-scan function.

All reported genomic positions are based on Human Genome Chromosome Build 37 (b37) and Human Genome ref. 19 (hg19) unless otherwise specified.

### Association

Quantile-Quantile (Q-Q) plots of the association P values were produced using the “qqman” R package, available at http://cran.r-project.org/web/packages/qqman/41. The genomic control (GC) factor, indicating inflation of positive P values, was calculated using PLINK.

PLINK was used to perform allelic association for each marker using Fisher’s exact test. Uncorrected P values, odds ratios, as well as 95% confidence intervals (95% CI) for the odds ratios were calculated. Bonferroni-corrected P values were also calculated. Genome-wide association results were visualized through Manhattan plots, generated using the R package “qqman”. Logistic regression with sex as a covariate was also performed using PLINK.

Association was assessed separately for all successfully genotyped OM patients (N = 803) versus controls (N = 2073), for only OM patients with recurrent disease (RAOM, N = 702) versus controls, and for chronic cases (COME, N = 512) versus controls.

LocusZoom v.1.1 (available at http://locuszoom.sph.umich.edu/locuszoom/42) was used to further visualize the association on chromosome 19 and show the position of each marker in relation to nearby genes.

Single markers showing extremely strong association with all OM/RAOM/COME were followed up but deemed likely artifacts, whereas signals with a vertical “smear”, indicating association to multiple genetic markers in close proximity, were deemed to be more plausible true signals. A nominal P value <5 × 10^−8^ was considered the threshold for genome-wide significance^27^.

### Validation

To validate the best results from the GWA study we genotyped the same 829 Finnish patients on another platform. As control we used 778 healthy Finnish blood donors. Genotyping was performed on the MassARRAY Platform from Sequenom (San Diego, USA) by the Institute for Molecular Medicine Finland FIMM Technology Centre, University of Helsinki. Three markers (rs4150992, rs16974263, rs268662; assay ID C__30608050_10, C__33845609_10, C___2928476_10) that failed assay design on Sequenom were genotyped by Taqman SNP Genotyping Assays (Life Technologies, Thermo Fisher Scientific Inc., Waltham, MA, USA). The variant rs885932 failed assay design on both Sequenom and Taqman. Odds ratios were calculated, and the two-tailed Fisher’s exact test was used to estimate P values. *P* < 0.05 was considered significant.

### Replication

4860 individuals from the UK were genotyped for rs16974263 and rs4150992. Assay design for rs268662 was attempted but failed. 1466 were cases (proband children with COME or their affected siblings), 3394 were set as unknown (parents and siblings without known COME).

The samples were genotyped using KASP primer extension sequencing by LGC Genomics (http://www.lgcgroup.com/products/kasp-genotyping-chemistry/). Individuals with more than one missing genotype were excluded from the analysis, as well as families showing inheritance errors in marker. PLINK was used to assess transmission disequilibrium (TDT) in the trios. P values, as well as ORs and OR 95% CIs were calculated.

The R package “metafor” (www.metafor-project.org^43^) was used to draw forestplots for visualization of odds ratios in each dataset.

### *In Silico* Analyses

Haploview v. 4.2 (http://www.broadinstitute.org/haploview/haploview^44^) was used to study linkage disequilibrium (LD) patterns on chromosome 19, and identify putative haplotype blocks in the region of the association signal. Visual inspection of pairwise D’values was used.

The UCSC Genome Browser at genome.ucsc.edu was used to look at the position of associated variants in relation to genes and specific exons, as well as to look at conservation patterns, likely functional elements, histone methylation, and expression patterns within the associated region on chromosome 19. The functional annotation of the mammalian genome 5 database (FANTOM5) at fantom.gsc.riken.jp, the GTEx Portal at www.gtexportal.org, and the Human Protein Atlas (www.proteinatlas.org) were used to examine the RNA and protein expression of the genes within the chromosome 19 region associated with OM.

We used the ToppGene portal (toppgene.cchmc.org) to prioritize candidate genes from our study by comparing them to a training dataset based on all previously described OM genes (as summarized in Hafrén *et al.*^10^), and including also the newly described *A2ML1* gene^19^. The ToppGene analysis retrieves information from a number of sources such as information on gene ontology (GO) categories (looking at which functional categories & pathways a set of genes fall into), phenotypes and diseases, pathways, previous publications, known interactions, etc. Each of the candidate genes is given a score based on their similarity to the genes in the training dataset, and an overall P value. WebGestalt, available at bioinfo.vanderbilt.edu/webgestalt^35^, was used to perform gene ontology analysis on all the known OM candidate genes, in addition to our six candidate genes.

## Additional Information

**How to cite this article**: Einarsdottir, E. *et al.* Genome-wide association analysis reveals variants on chromosome 19 that contribute to childhood risk of chronic otitis media with effusion. *Sci. Rep.* **6**, 33240; doi: 10.1038/srep33240 (2016).

## Supplementary Material

Supplementary Information

## Figures and Tables

**Table 1:** The top results (A) in the GWA study on Finnish OM children, (B) the validation on another platform (Sequenom or Taqman), and (C) the replication data in an independent UK cohort.

Variant	Chromosome	Position (b37)	Risk allele	A		B			C	
				GWA results in Finnish index cohort		Validation on another platform			Replication in UK cohort	
				OR	P value	OR	P value	Concordance between A and B	OR	P value
rs3821170	2	207453310	G	2.44	2.83 × 10^−10^	1.32	0.0456^+^	0.92	NA	NA
rs885932	6	29837507	A	1.78	2.58 × 10^−10^	failed assay design		NA	NA	NA
rs4803329	19	40877284	A	1.44	9.63 × 10^−5^	NA	NA	NA	NA	NA
rs16974263	19	40913539	A	1.59	1.77 × 10^−7^	1.41	0.0068*	1.00	0.72	0.0003
rs268662	19	40923375	C	1.54	1.56 × 10^−6^	1.63	<0.0001*	0.97	failed assay design	
rs4150992	19	40928944	G	1.52	3.37 × 10^−6^	1.38	0.0083*	1.00	0.71	0.0002
rs2406176	21	19719426	A	2.70	1.72 × 10^−30^	1.08	0.3683^+^	0.73	NA	NA
rs4825724	X	119868403	A	2.28	1.37 × 10^−22^	1.05	0.5247^+^	0.69	NA	NA

Concordance is calculated on genotyped cases in the original GWA data and in the validation data.

^+^ = Genotyping on Sequenom platform.

* = Genotyping on Taqman platform.

**Table 2:** Finnish study subjects Finnish study populations and their clinical characteristics.

Cohort	Helsinki OM cohort		Tympanostomy vs tympanostomy + adenoidectomy cohort		Total	
	N = 601		N = 202		N = 803	
	N	%	N	%	N	%
Male	363	60%	109	54%	472	59%
COME	411	68%	101	50%	512	64%
RAOM	520	87%	182	90%	702	87%
Adenoidectomy	394	66%	101	50%	495	62%
Tympanostomy tubes	548	91%	202	100%	750	93%
Multiple tympanostomy tubes	192	32%	53	26%	245	31%

COME = Chronic otitis media with effusion, RAOM = Recurrent acute otitis media.

## References

[b1] Freid V. M., Makuc D. M. & Rooks R. N. Ambulatory health care visits by children: principal diagnosis and place of visit. *Vital and health statistics. Series 13*, Data from the National Health Survey, 1–23 (1998). 9631643

[b2] Gonzales R., Malone D. C., Maselli J. H. & Sande M. A. Excessive antibiotic use for acute respiratory infections in the United States. Clinical infectious diseases: an official publication of the Infectious Diseases Society of America 33, 757–762, doi: 10.1086/322627 (2001). 11512079

[b3] Rovers M. M. The burden of otitis media. Vaccine 26 Suppl 7, G2–G4, doi: 10.1016/j.vaccine.2008.11.005 (2008). 19094933

[b4] Jotic A. *et al.* Polymorphisms in Toll-like receptors 2 and 4 genes and their expression in chronic suppurative otitis media. Auris, nasus, larynx 42, 431–437, doi: 10.1016/j.anl.2015.04.010 (2015). 26055429

[b5] Wiertsema S. P. *et al.* Association of CD14 promoter polymorphism with otitis media and pneumococcal vaccine responses. Clinical and vaccine immunology: CVI 13, 892–897, doi: 10.1128/CVI.00100-06 (2006). 16893989 PMC1539116

[b6] Patel J. A. *et al.* Association of proinflammatory cytokine gene polymorphisms with susceptibility to otitis media. Pediatrics 118, 2273–2279, doi: 10.1542/peds.2006-0764 (2006). 17142509

[b7] Emonts M. *et al.* Genetic polymorphisms in immunoresponse genes TNFA, IL6, IL10, and TLR4 are associated with recurrent acute otitis media. Pediatrics 120, 814–823, doi: 10.1542/peds.2007-0524 (2007). 17908769

[b8] Daly K. A. *et al.* Chronic and recurrent otitis media: a genome scan for susceptibility loci. American journal of human genetics 75, 988–997, doi: 10.1086/426061 (2004). 15514890 PMC1225283

[b9] Gentile D. A. *et al.* Cytokine gene polymorphisms moderate illness severity in infants with respiratory syncytial virus infection. Human immunology 64, 338–344 (2003). 12590978 10.1016/s0198-8859(02)00827-3

[b10] Hafren L., Kentala E., Einarsdottir E., Kere J. & Mattila P. S. Current knowledge of the genetics of otitis media. Current allergy and asthma reports 12, 582–589, doi: 10.1007/s11882-012-0292-1 (2012). 22886440

[b11] Nuytinck L., De Meester E., Van Thielen M. & Govaerts P. Role of mannose-binding lectin (MBL2) genotyping in predicting the risk of recurrent otitis media (rOM). Advances in experimental medicine and biology 586, 281–290, doi: 10.1007/0-387-34134-X_19 (2006). 16893079

[b12] Wiertsema S. P. *et al.* Functional polymorphisms in the mannan-binding lectin 2 gene: effect on MBL levels and otitis media. The Journal of allergy and clinical immunology 117, 1344–1350, doi: 10.1016/j.jaci.2006.01.031 (2006). 16750996

[b13] Hafren L. *et al.* Predisposition to Childhood Otitis Media and Genetic Polymorphisms within the Toll-Like Receptor 4 (TLR4) Locus. PloS one 10, e0132551, doi: 10.1371/journal.pone.0132551 (2015). 26177520 PMC4503307

[b14] MacArthur C. J. *et al.* Genetic susceptibility to chronic otitis media with effusion: candidate gene single nucleotide polymorphisms. The Laryngoscope 124, 1229–1235, doi: 10.1002/lary.24349 (2014). 23929584 PMC3971016

[b15] Sale M. M. *et al.* Evaluation of 15 functional candidate genes for association with chronic otitis media with effusion and/or recurrent otitis media (COME/ROM). PloS one 6, e22297, doi: 10.1371/journal.pone.0022297 (2011). 21857919 PMC3156706

[b16] Alper C. M., Winther B., Hendley J. O. & Doyle W. J. Cytokine polymorphisms predict the frequency of otitis media as a complication of rhinovirus and RSV infections in children. European archives of oto-rhino-laryngology: official journal of the European Federation of Oto-Rhino-Laryngological Societies 266, 199–205, doi: 10.1007/s00405-008-0729-2 (2009). PMC7087847 18560870

[b17] Revai K. *et al.* Association between cytokine gene polymorphisms and risk for upper respiratory tract infection and acute otitis media. Clinical infectious diseases: an official publication of the Infectious Diseases Society of America 49, 257–261, doi: 10.1086/599833 (2009). 19522649 PMC2759686

[b18] Ilia S., Goulielmos G. N., Samonis G. & Galanakis E. Polymorphisms in IL-6, IL-10, TNF-alpha, IFN-gamma and TGF-beta1 genes and susceptibility to acute otitis media in early infancy. The Pediatric infectious disease journal 33, 518–521, doi: 10.1097/INF.0000000000000229 (2014). 24463810

[b19] Nokso-Koivisto J. *et al.* Polymorphisms of immunity genes and susceptibility to otitis media in children. PloS one 9, e93930, doi: 10.1371/journal.pone.0093930 (2014). 24718616 PMC3981756

[b20] Joki-Erkkila V. P., Puhakka H. & Hurme M. Cytokine gene polymorphism in recurrent acute otitis media. Archives of otolaryngology–head & neck surgery 128, 17–20 (2002). 11784248 10.1001/archotol.128.1.17

[b21] McCormick M. E., Sheyn A., Haupert M., Thomas R. & Folbe A. J. Predicting complications after adenotonsillectomy in children 3 years old and younger. International journal of pediatric otorhinolaryngology 75, 1391–1394, doi: 10.1016/j.ijporl.2011.07.035 (2011). 21889216

[b22] Patel A., Gentile D. A., Koehrsen J. & Skoner D. P. Association Between TGF-B1 Genotype and the Development of Otitis Media (OM) in Young Children during Respiratory Virus Season. The Journal of allergy and clinical immunology 117, 1 (2005).

[b23] Rye M. S. *et al.* FBXO11, a regulator of the TGFbeta pathway, is associated with severe otitis media in Western Australian children. Genes and immunity 12, 352–359, doi: 10.1038/gene.2011.2 (2011). 21293382

[b24] Segade F. *et al.* Association of the FBXO11 gene with chronic otitis media with effusion and recurrent otitis media: the Minnesota COME/ROM Family Study. Archives of otolaryngology–head & neck surgery 132, 729–733, doi: 10.1001/archotol.132.7.729 (2006). 16847180 PMC1904347

[b25] Ubell M. L., Khampang P. & Kerschner J. E. Mucin gene polymorphisms in otitis media patients. The Laryngoscope 120, 132–138, doi: 10.1002/lary.20688 (2010). 19718741 PMC2919485

[b26] Schmuczerova J., Brdicka R., Dostal M., Sram R. J. & Topinka J. Genetic variability of HVRII mtDNA in cord blood and respiratory morbidity in children. Mutation research 666, 1–7, doi: 10.1016/j.mrfmmm.2009.03.002 (2009). 19481673

[b27] Barsh G. S., Copenhaver G. P., Gibson G. & Williams S. M. Guidelines for genome-wide association studies. PLoS genetics 8, e1002812, doi: 10.1371/journal.pgen.1002812 (2012). 22792080 PMC3390399

[b28] Bhutta M. F. Epidemiology and pathogenesis of otitis media: construction of a phenotype landscape. Audiology & neuro-otology 19, 210–223, doi: 10.1159/000358549 (2014). 24819621

[b29] Zucchelli M. *et al.* PepT1 oligopeptide transporter (SLC15A1) gene polymorphism in inflammatory bowel disease. Inflammatory bowel diseases 15, 1562–1569, doi: 10.1002/ibd.20963 (2009). 19462432

[b30] Zhang G. *et al.* Opposite gene by environment interactions in Karelia for CD14 and CC16 single nucleotide polymorphisms and allergy. Allergy 64, 1333–1341, doi: 10.1111/j.1398-9995.2009.02006.x (2009). 19222419

[b31] Hancock D. G. *et al.* A systems biology approach to the analysis of subset-specific responses to lipopolysaccharide in dendritic cells. PloS one 9, e100613, doi: 10.1371/journal.pone.0100613 (2014). 24949855 PMC4065045

[b32] Chen W. M. *et al.* Significant linkage at chromosome 19q for otitis media with effusion and/or recurrent otitis media (COME/ROM). BMC medical genetics 12, 124, doi: 10.1186/1471-2350-12-124 (2011). 21943191 PMC3191346

[b33] Hammaren-Malmi S., Tarkkanen J. & Mattila P. S. Analysis of risk factors for childhood persistent middle ear effusion. Acta oto-laryngologica 125, 1051–1054, doi: 10.1080/00016480510038040 (2005). 16298785

[b34] Jasenosky L. D., Scriba T. J., Hanekom W. A. & Goldfeld A. E. T cells and adaptive immunity to Mycobacterium tuberculosis in humans. Immunological reviews 264, 74–87, doi: 10.1111/imr.12274 (2015). 25703553

[b35] Sun J. & Braciale T. J. Role of T cell immunity in recovery from influenza virus infection. Current opinion in virology 3, 425–429, doi: 10.1016/j.coviro.2013.05.001 (2013). 23721865 PMC3804899

[b36] Sung C. O., Song I. H. & Sohn I. A distinctive ovarian cancer molecular subgroup characterized by poor prognosis and somatic focal copy number amplifications at chromosome 19. Gynecologic oncology 132, 343–350, doi: 10.1016/j.ygyno.2013.11.036 (2014). 24321399

[b37] Goycoolea M. V., Hueb M. M. & Ruah C. Otitis media: the pathogenesis approach. Definitions and terminology. Otolaryngologic clinics of North America 24, 757–761 (1991). 1870869

[b38] Kristiansson K. *et al.* Genome-wide screen for metabolic syndrome susceptibility Loci reveals strong lipid gene contribution but no evidence for common genetic basis for clustering of metabolic syndrome traits. Circulation. Cardiovascular genetics 5, 242–249, doi: 10.1161/CIRCGENETICS.111.961482 (2012). 22399527 PMC3378651

[b39] Howie B. N., Donnelly P. & Marchini J. A flexible and accurate genotype imputation method for the next generation of genome-wide association studies. PLoS genetics 5, e1000529, doi: 10.1371/journal.pgen.1000529 (2009). 19543373 PMC2689936

[b40] Purcell S. *et al.* PLINK: a tool set for whole-genome association and population-based linkage analyses. American journal of human genetics 81, 559–575, doi: 10.1086/519795 (2007). 17701901 PMC1950838

[b41] Turner S. qqman: an R package for visualizing GWAS results using Q-Q and manhattan plots. biorXiv, doi: 10.1101/005165 (2014).

[b42] Pruim R. J. *et al.* LocusZoom: regional visualization of genome-wide association scan results. Bioinformatics 26, 2336–2337, doi: 10.1093/bioinformatics/btq419 (2010). 20634204 PMC2935401

[b43] Viechtbauer W. Conducting meta-analyses in R with the metafor package. Journal of Statistical Software 36(3), 1–48 (2010).

[b44] Barrett J. C., Fry B., Maller J. & Daly M. J. Haploview: analysis and visualization of LD and haplotype maps. Bioinformatics 21, 263–265, doi: 10.1093/bioinformatics/bth457 (2005). 15297300

