# Supplementary material for: Genome-wide association analysis reveals variants on chromosome 19 that contribute to childhood risk of chronic otitis media with effusion
Source: Sci Rep. 2016 Sep 16;6:33240. doi: 10.1038/srep33240 (PMC5025747; doi:10.1038/srep33240)
Supplement: Supplementary Information [file srep33240-s1.pdf]

Supplementary data

for "*Genome-wide association analysis reveals variants on chromosome 19 that contribute to childhood risk of chronic otitis media with effusion*" by

Elisabet Einarsdottir (1,2,5)* & Lena Hafrén (1,3,5), Eira Leinonen (1), Mahmood F. Bhutta (4), Erna Kentala (3), Juha Kere (1,2), Petri S. Mattila (3)

1 - Folkhälsan Institute of Genetics, and Molecular Neurology Research Program, University of Helsinki, Helsinki, Finland
2 - Department of Biosciences and Nutrition, Karolinska Institutet, Huddinge, Sweden
3 - Department of Otorhinolaryngology, Helsinki University Hospital, Helsinki, Finland
4 - Children's Surgical Centre, Phnom Penh, Cambodia
5 - Shared first authors
* Corresponding author

Supplementary Table 1
Summary of association to the three most highly significant markers on the chromosome 19 in the GWA study, validation and replication.

Supplementary Table 2
GWA association with P values adjusted for multiple testing through several methods (as implemented in the PLINK function --adjust). The top 50 associated markers are shown for OM all affected, COME and RAOM.

Supplementary Table 3
Results of the ToppGene analysis to rank the six candidate genes. The average score and overall P value for the likelihood of each gene being involved in OM are shown. (toppgene.cchmc.org)

Supplementary Table 4
A summary of previously published associations to otitis media or related phenotypes. Association in the current GWA study to those specific markers/genomic location is also shown.

Supplementary Figure 1
Q-Q plots of the expected vs. observed distribution of p-values in the GWA study of All OM (A), COME (B), and RAOM (C).

Supplementary Figure 2

Association to OM (all affected) in markers within a 400 kb region surrounding rs3821170 (A), rs885932 (B), rs2406176 (C), and rs4825724 as well as their estimated LD with each other. The genes that are located within the region are shown, as well as recombination patterns (plotted based on data from the hg19/1000 Genomes dataset, Nov 2014 EUR population).

Supplementary Figure 3

Pairwise r^2 LD between all markers in the region based on our data. Our associated markers constitute a block of strong LD. The region shown is the same as in Figure 2C.

Supplementary Figure 4

GO analysis of previously known OM genes showing their enrichment in biological process, molecular function, and cellular component. The output is presented as a figure, as well as in table format.

Supplementary Table 1 - Summary of association to three most highly associated markers on chr 19 in GWA study, validation and replication in succesfully genotyped study subjects

rs268662

group	N	OR	95% CI	P value
GWAS, all affected	803	1.54	1.29-1.83	1.56x10^{-6}
GWAS, RAOM	702	1.58	1.32-1.91	8.38x10^{-7}
GWAS, COME	512	1.73	1.42-2.12	6.52x10^{-8}
Validation, all affected	829	1.63	1.30-2.04	< 0.0001
Validation, RAOM	721	1.52	1.22-1.89	0.0002
Validation, COME	524	1.76	1.39-2.21	< 0.0001
UK replication, COME	1247 trios			

rs16974263

group	OR	95% CI	P value
GWAS, all affected	1.59	1.33-1.89	1.77x10^{-7}
GWAS, RAOM	1.64	1.36-1.96	1.02x10^{-7}
GWAS, COME	1.75	1.43-2.14	2.92x10^{-8}
Validation, all affected	1.41	1.10-1.79	0.0068
Validation, RAOM	1.45	1.14-1.84	0.003
Validation, COME	1.41	1.09-1.83	0.0099
UK replication, COME	0.72	0.60-0.86	0.00032

rs4150992

group	OR	95% CI	P value
GWAS, all affected	1.52	1.27-1.81	3.37x10^{-6}
GWAS, RAOM	1.55	1.29-1.87	2.67x10^{-6}
GWAS, COME	1.65	1.35-2.02	1.03x10^{-6}
Validation, all affected	1.38	1.09-1.74	0.0083
Validation, RAOM	1.32	1.05-1.68	0.0217
Validation, COME	1.48	1.15-1.90	0.0024
UK replication, COME	0.71	0.59-0.85	0.00016

Supplementary Table 2

GWA association with P values adjusted for multiple testing through several methods (as implemented in the PLINK function --adjust). The top 50 associated markers are shown for OM all affected, COME and RAOM.

All OM affected

CHR	SNP	UNADJ	GC	BONF	HOLM	SIDAK_SS	SIDAK_SD	FDR_BH	FDR_BY
21	rs2406176	1.724e-30	1.031e-29	5.512e-25	5.512e-25	INF	INF	5.512e-25	7.304e-24
X	rs4825724	1.37e-22	5.041e-22	4.38e-17	4.38e-17	INF	INF	2.19e-17	2.902e-16
6	rs885932	2.581e-10	4.481e-10	8.25e-05	8.25e-05	8.25e-05	8.25e-05	2.264e-05	0.0003001
2	rs3821170	2.833e-10	4.907e-10	9.057e-05	9.056e-05	9.056e-05	9.056e-05	2.264e-05	0.0003001
19	rs16974263	1.768e-07	2.586e-07	0.05651	0.05651	0.05494	0.05494	0.0113	0.1498
19	rs268662	1.564e-06	2.161e-06	0.4999	0.4999	0.3934	0.3934	0.08331	1
9	rs649057	1.971e-06	2.708e-06	0.6301	0.6301	0.4675	0.4675	0.08875	1
X	rs7891968	2.221e-06	3.042e-06	0.71	0.71	0.5084	0.5084	0.08875	1
6	rs17077968	2.831e-06	3.853e-06	0.9051	0.9051	0.5955	0.5955	0.1006	1
19	rs4150992	3.365e-06	4.558e-06	1	1	0.6589	0.6589	0.1076	1
19	rs7254075	3.887e-06	5.247e-06	1	1	0.7114	0.7114	0.113	1
20	rs6018337	1.136e-05	1.491e-05	1	1	0.9735	0.9735	0.3026	1
5	rs884252	1.335e-05	1.745e-05	1	1	0.986	0.986	0.3282	1
5	rs752921	1.469e-05	1.916e-05	1	1	0.9909	0.9909	0.3354	1
10	rs7092964	1.621e-05	2.109e-05	1	1	0.9944	0.9944	0.3456	1
6	rs7748283	2.442e-05	3.143e-05	1	1	0.9996	0.9996	0.4878	1
9	rs7018886	2.796e-05	3.587e-05	1	1	0.9999	0.9999	0.5022	1
7	rs2006644	3.026e-05	3.874e-05	1	1	0.9999	0.9999	0.5022	1
13	rs10507724	3.337e-05	4.261e-05	1	1	1	1	0.5022	1
9	rs6477122	3.384e-05	4.32e-05	1	1	1	1	0.5022	1
9	rs10781321	3.524e-05	4.494e-05	1	1	1	1	0.5022	1
8	rs6601627	3.6e-05	4.588e-05	1	1	1	1	0.5022	1
1	rs10907223	3.809e-05	4.847e-05	1	1	1	1	0.5022	1
9	rs7849878	4.722e-05	5.975e-05	1	1	1	1	0.5022	1
12	rs10876062	4.801e-05	6.074e-05	1	1	1	1	0.5022	1
9	rs634202	5.142e-05	6.494e-05	1	1	1	1	0.5022	1
1	rs950327	5.183e-05	6.543e-05	1	1	1	1	0.5022	1
3	rs12634070	5.456e-05	6.88e-05	1	1	1	1	0.5022	1
3	rs3772728	5.566e-05	7.014e-05	1	1	1	1	0.5022	1
4	rs13141555	5.592e-05	7.047e-05	1	1	1	1	0.5022	1
19	rs8112673	5.662e-05	7.132e-05	1	1	1	1	0.5022	1
9	rs10512052	5.736e-05	7.223e-05	1	1	1	1	0.5022	1
5	rs2242132	5.775e-05	7.271e-05	1	1	1	1	0.5022	1
3	rs1073231	5.808e-05	7.311e-05	1	1	1	1	0.5022	1
9	rs10960736	5.864e-05	7.38e-05	1	1	1	1	0.5022	1
8	rs2513924	6.117e-05	7.691e-05	1	1	1	1	0.5022	1
6	rs2063345	6.142e-05	7.72e-05	1	1	1	1	0.5022	1
18	rs9951080	6.636e-05	8.325e-05	1	1	1	1	0.5022	1
6	rs670369	6.945e-05	8.702e-05	1	1	1	1	0.5022	1
9	rs7033444	7.292e-05	9.126e-05	1	1	1	1	0.5022	1
10	rs2299939	7.516e-05	9.399e-05	1	1	1	1	0.5022	1
8	rs9314561	7.547e-05	9.437e-05	1	1	1	1	0.5022	1
8	rs7823866	7.661e-05	9.576e-05	1	1	1	1	0.5022	1
13	rs5007254	7.837e-05	9.79e-05	1	1	1	1	0.5022	1
5	rs803218	7.854e-05	9.81e-05	1	1	1	1	0.5022	1
16	rs247615	7.975e-05	9.958e-05	1	1	1	1	0.5022	1
5	rs1842226	8.399e-05	0.0001047	1	1	1	1	0.5022	1
7	rs1177935	8.46e-05	0.0001055	1	1	1	1	0.5022	1
13	rs9556369	8.555e-05	0.0001066	1	1	1	1	0.5022	1
6	rs723587	8.599e-05	0.0001072	1	1	1	1	0.5022	1

COME

CHR	SNP	UNADJ	GC	BONF	HOLM	SIDAK_SS	SIDAK_SD	FDR_BH	FDR_BY
X	rs4825724	7.273e-18	1.569e-17	2.325e-12	2.325e-12	INF	INF	2.325e-12	3.081e-11
21	rs2406176	1.593e-17	3.381e-17	5.091e-12	5.091e-12	INF	INF	2.546e-12	3.374e-11
19	rs16974263	2.924e-08	4.045e-08	0.009348	0.009348	0.009305	0.009305	0.003116	0.0413
19	rs268662	6.521e-08	8.877e-08	0.02085	0.02084	0.02063	0.02063	0.005211	0.06906
2	rs3821170	6.18e-07	8.046e-07	0.1976	0.1976	0.1793	0.1793	0.03496	0.4633
9	rs649057	6.561e-07	8.533e-07	0.2097	0.2097	0.1892	0.1892	0.03496	0.4633
19	rs4150992	1.03e-06	1.327e-06	0.3292	0.3292	0.2805	0.2805	0.04697	0.6225
6	rs885932	1.211e-06	1.556e-06	0.3872	0.3872	0.321	0.321	0.04697	0.6225
19	rs9917042	1.322e-06	1.696e-06	0.4228	0.4228	0.3448	0.3448	0.04697	0.6225
19	rs4803329	2.679e-06	3.388e-06	0.8563	0.8563	0.5753	0.5753	0.08563	1
8	rs2513924	1.007e-05	1.241e-05	1	1	0.9601	0.9601	0.2928	1
3	rs3772728	1.272e-05	1.56e-05	1	1	0.9829	0.9829	0.3347	1
12	rs7972426	1.521e-05	1.86e-05	1	1	0.9923	0.9923	0.3347	1
3	rs1073231	1.568e-05	1.915e-05	1	1	0.9933	0.9933	0.3347	1
4	rs640246	1.598e-05	1.952e-05	1	1	0.994	0.994	0.3347	1
X	rs5909600	1.675e-05	2.044e-05	1	1	0.9953	0.9953	0.3347	1
16	rs735820	1.88e-05	2.288e-05	1	1	0.9975	0.9975	0.3535	1
7	rs978897	2.262e-05	2.744e-05	1	1	0.9993	0.9993	0.3841	1
1	rs2335406	2.69e-05	3.251e-05	1	1	0.9998	0.9998	0.3841	1
20	rs157640	2.712e-05	3.278e-05	1	1	0.9998	0.9998	0.3841	1
20	rs530008	2.732e-05	3.302e-05	1	1	0.9998	0.9998	0.3841	1
6	rs17840238	2.784e-05	3.364e-05	1	1	0.9999	0.9999	0.3841	1
16	rs16957355	2.855e-05	3.448e-05	1	1	0.9999	0.9999	0.3841	1
7	rs2006644	2.981e-05	3.596e-05	1	1	0.9999	0.9999	0.3841	1
X	rs7391585	3.233e-05	3.895e-05	1	1	1	1	0.3841	1
4	rs12499725	3.236e-05	3.898e-05	1	1	1	1	0.3841	1
9	rs7849878	3.373e-05	4.059e-05	1	1	1	1	0.3841	1
5	rs803218	3.736e-05	4.487e-05	1	1	1	1	0.3841	1
3	rs9843963	3.778e-05	4.537e-05	1	1	1	1	0.3841	1
10	rs2489382	3.847e-05	4.619e-05	1	1	1	1	0.3841	1
3	rs12634070	3.906e-05	4.688e-05	1	1	1	1	0.3841	1
12	rs1017301	4.01e-05	4.81e-05	1	1	1	1	0.3841	1
16	rs7202727	4.016e-05	4.817e-05	1	1	1	1	0.3841	1
7	rs1950148	4.085e-05	4.899e-05	1	1	1	1	0.3841	1
8	rs9642980	4.45e-05	5.327e-05	1	1	1	1	0.391	1
16	rs1560104	4.605e-05	5.509e-05	1	1	1	1	0.391	1
6	rs17077968	5.152e-05	6.15e-05	1	1	1	1	0.391	1
5	rs884252	5.155e-05	6.154e-05	1	1	1	1	0.391	1
16	rs2865582	5.272e-05	6.291e-05	1	1	1	1	0.391	1
8	rs6980766	5.344e-05	6.374e-05	1	1	1	1	0.391	1
14	rs4905781	5.356e-05	6.388e-05	1	1	1	1	0.391	1
3	rs10935794	5.543e-05	6.608e-05	1	1	1	1	0.391	1
5	rs6891672	5.546e-05	6.611e-05	1	1	1	1	0.391	1
1	rs12033775	5.684e-05	6.772e-05	1	1	1	1	0.391	1
6	rs1496117	5.7e-05	6.79e-05	1	1	1	1	0.391	1
5	rs832540	5.789e-05	6.895e-05	1	1	1	1	0.391	1
4	rs885395	5.857e-05	6.975e-05	1	1	1	1	0.391	1
19	rs7254075	5.91e-05	7.036e-05	1	1	1	1	0.391	1
16	rs7188223	6.278e-05	7.465e-05	1	1	1	1	0.391	1
15	rs1025768	6.411e-05	7.621e-05	1	1	1	1	0.391	1

RAOM

CHR	SNP	UNADJ	GC	BONF	HOLM	SIDAK_SS	SIDAK_SD	FDR_BH	FDR_BY
21	rs2406176	2.457e-28	1.434e-27	7.854e-23	7.854e-23	INF	INF	7.854e-23	1.041e-21
X	rs4825724	2.264e-20	7.841e-20	7.237e-15	7.237e-15	INF	INF	3.618e-15	4.795e-14
6	rs885932	1.818e-12	3.762e-12	5.812e-07	5.812e-07	5.812e-07	5.812e-07	1.937e-07	2.567e-06
2	rs3821170	1.023e-08	1.661e-08	0.003271	0.003271	0.003265	0.003265	0.0008176	0.01084
19	rs16974263	1.023e-07	1.557e-07	0.03269	0.03269	0.03216	0.03216	0.006538	0.08664
19	rs268662	8.383e-07	1.204e-06	0.268	0.268	0.2351	0.2351	0.04466	0.5919
8	rs9314561	2.643e-06	3.677e-06	0.8449	0.8449	0.5704	0.5704	0.09844	1
19	rs4150992	2.667e-06	3.71e-06	0.8526	0.8526	0.5737	0.5737	0.09844	1
6	rs17077968	2.771e-06	3.851e-06	0.886	0.886	0.5877	0.5877	0.09844	1
X	rs7891968	5.658e-06	7.707e-06	1	1	0.8361	0.8361	0.1809	1
6	rs2063345	9.95e-06	1.335e-05	1	1	0.9584	0.9584	0.2892	1
5	rs1363268	1.368e-05	1.819e-05	1	1	0.9874	0.9874	0.3644	1
10	rs7092964	1.783e-05	2.353e-05	1	1	0.9966	0.9966	0.4383	1
20	rs6018337	2.048e-05	2.693e-05	1	1	0.9986	0.9986	0.4677	1
1	rs10907223	2.204e-05	2.893e-05	1	1	0.9991	0.9991	0.4698	1
2	rs10171238	2.363e-05	3.095e-05	1	1	0.9995	0.9995	0.4702	1
8	rs6601627	2.594e-05	3.389e-05	1	1	0.9997	0.9997	0.4702	1
6	rs9382618	2.815e-05	3.669e-05	1	1	0.9999	0.9999	0.4702	1
5	rs888804	2.849e-05	3.712e-05	1	1	0.9999	0.9999	0.4702	1
19	rs7254075	2.942e-05	3.83e-05	1	1	0.9999	0.9999	0.4702	1
4	rs17510449	3.331e-05	4.322e-05	1	1	1	1	0.5023	1
9	rs7849878	3.516e-05	4.555e-05	1	1	1	1	0.5023	1
6	rs6939922	3.728e-05	4.822e-05	1	1	1	1	0.5023	1
18	rs10502503	3.771e-05	4.876e-05	1	1	1	1	0.5023	1
5	rs6860141	4.246e-05	5.473e-05	1	1	1	1	0.5199	1
6	rs7748283	4.473e-05	5.757e-05	1	1	1	1	0.5199	1
7	rs1177935	4.793e-05	6.157e-05	1	1	1	1	0.5199	1
5	rs2242132	4.815e-05	6.184e-05	1	1	1	1	0.5199	1
10	rs880272	4.839e-05	6.215e-05	1	1	1	1	0.5199	1
5	rs884252	4.881e-05	6.267e-05	1	1	1	1	0.5199	1
18	rs4800827	5.172e-05	6.63e-05	1	1	1	1	0.5199	1
3	rs9858935	5.3e-05	6.79e-05	1	1	1	1	0.5199	1
6	rs6933713	5.874e-05	7.504e-05	1	1	1	1	0.5199	1
7	rs2307252	5.894e-05	7.529e-05	1	1	1	1	0.5199	1
18	rs9951080	5.895e-05	7.53e-05	1	1	1	1	0.5199	1
1	rs950327	6.2e-05	7.909e-05	1	1	1	1	0.5199	1
5	rs752921	6.233e-05	7.95e-05	1	1	1	1	0.5199	1
12	rs10876062	6.31e-05	8.045e-05	1	1	1	1	0.5199	1
3	rs3772728	6.531e-05	8.319e-05	1	1	1	1	0.5199	1
3	rs878382	6.567e-05	8.364e-05	1	1	1	1	0.5199	1
9	rs649057	6.679e-05	8.503e-05	1	1	1	1	0.5199	1
13	rs10507724	6.896e-05	8.771e-05	1	1	1	1	0.5199	1
5	rs4596407	7.265e-05	9.227e-05	1	1	1	1	0.5199	1
11	rs747784	7.28e-05	9.246e-05	1	1	1	1	0.5199	1
X	rs5983012	7.42e-05	9.418e-05	1	1	1	1	0.5199	1
11	rs747783	7.64e-05	9.69e-05	1	1	1	1	0.5199	1
11	rs1484425	7.643e-05	9.694e-05	1	1	1	1	0.5199	1
3	rs2365835	8.006e-05	0.0001014	1	1	1	1	0.5332	1
7	rs2006644	8.207e-05	0.0001039	1	1	1	1	0.5354	1
1	rs1572968	8.517e-05	0.0001077	1	1	1	1	0.5446	1

Supplementary Table 3

The table shows the results of the ToppGene analysis to rank the six candidate genes. The average score and overall P value for the likelihood of each gene being involved in OM are shown. (toppgene.cchmc.org)

Rank	1	2	3	4	5	6
**GeneSymbol**	**PLD3**	**BLVRB**	**SERTAD1**	**PRX**	**SERTAD3**	**HIPK4**
GeneID	23646	645	29950	57716	29946	147746
GO: Molecular Function Score	0,0334	0,0000	0,0334	0,0334	0,0334	0,2411
GO: Molecular Function P value	0,1714	0,6113	0,1714	0,1714	0,1714	0,1104
GO: Biological Process Score	0,9680	0,7166	1,0000	0,9999	1,0000	0,9992
GO: Biological Process P value	0,1880	0,2333	0,0842	0,1156	0,0988	0,1326
GO: Cellular Component Score	0,8775	0,8581	0,1754	0,3410	0,1754	0,1754
GO: Cellular Component P value	0,0451	0,0500	0,2213	0,1826	0,2213	0,2213
Human Phenotype Score	-1,0000	-1,0000	-1,0000	0,3341	-1,0000	-1,0000
Human Phenotype P value	1,0000	1,0000	1,0000	0,0124	1,0000	1,0000
Mouse Phenotype Score	-1,0000	-1,0000	0,9763	1,0000	-1,0000	-1,0000
Mouse Phenotype P value	1,0000	1,0000	0,0680	0,0354	1,0000	1,0000
Pathway Score	0,0000	0,6251	-1,0000	-1,0000	-1,0000	-1,0000
Pathway P value	0,5252	0,0238	1,0000	1,0000	1,0000	1,0000
Pubmed Score	0,2823	0,0000	0,0000	0,0000	0,1890	0,0000
Pubmed P value	0,1210	0,5709	0,5709	0,5709	0,1352	0,5709
Interaction Score	0,4292	0,0000	0,7412	0,0000	0,0000	0,0000
Interaction P value	0,0378	0,5242	0,0167	0,5242	0,5242	0,5242
Coexpression Score	0,9263	0,8235	0,8495	0,4480	0,8435	0,0000
Coexpression P value	0,0339	0,0521	0,0467	0,1234	0,0478	0,6005
Coexpression Atlas Score	0,7534	0,4321	0,2373	0,0000	0,0000	-1,0000
Coexpression Atlas P value	0,0287	0,0503	0,0680	0,5418	0,5418	1,0000
Drug Score	1,0000	1,0000	0,9844	1,0000	0,8957	0,0770
Drug P value	0,0600	0,0600	0,1431	0,0740	0,1824	0,2856
Disease Score	0,0000	0,0766	0,0000	0,0000	-1,0000	-1,0000
Disease P value	0,5246	0,0424	0,5246	0,5246	1,0000	1,0000
**Average Score**	**0,5286**	**0,4662**	**0,5005**	**0,3779**	**0,3985**	**0,2419**
**Overall P value**	**0,0281**	**0,0398**	**0,0444**	**0,0580**	**0,3278**	**0,7282**

Supplementary Table 4

Marker	Association in current GWAS	chr	Position (b37)	Location/gene	Reference	
rs1175549	in gwas, p = 0.6	1	3691727	CCDC27	Rye et al 2012	p=2.65x10-5
rs12728900	in gwas, p = 0.15	1	26746807	LIN28	Rye et al 2012	p=2.28x10-5
rs1801274	in gwas, p = 0.81	1	161479745	exon of FCGR2A	Wiertsema et al, 2006b	p<0.05
rs3024509	nothing <0.01 within 10k	1	206943297	IL10	Sale et al 2008	
rs1554286	nothing <0.01 within 10k	1	206944233	intron of IL10	Sale et al 2008	
rs3021094	in gwas, p = 0.87	1	206944952	intron of IL10	Sale et al 2008	
rs1800872	nothing <0.01 within 10k	1	206946407	promoter of IL10	Alper et at 2009	p<0.05
rs1800871	nothing <0.01 within 10k	1	206946634	promoter of IL10	Alper et at 2009	p<0.05
rs1800896	in gwas, p = 0.57	1	206946897	promoter of IL10	Emonts et al 2007b	
rs1800893	nothing <0.01 within 10k	1	206947167	promoter of IL10	Sale et al 2008	
rs1800890	nothing <0.01 within 10k	1	206949365	promoter of IL10	Sale et al 2008	
rs13408922	in gwas, p = 0.27	2	31444826	CAPN14	Rye et al 2012	p=1.32x10-6
rs13386745	rs13386968 p = 0.0054	2	31445615	CAPN14	Rye et al 2012	p=1.63x10-6
rs13386850	in gwas, p = 0.36	2	31445691	CAPN14	Rye et al 2012	p=1.32x10-6
rs2020911	nothing <0.01 within 10k	2	48030838	intron of MSH6	Segade et al 2006	p=0.055
rs3136367	nothing <0.01 within 10k	2	48033551	intron of MSH6	Segade et al 2006	p=0.087
rs330787	in gwas, p = 0.57	2	48041377	intron of FBXO11	Rye et al 2011	
rs2134056	nothing <0.01 within 10k	2	48073117	intron of FBXO11	Segade et al 2006	p<0.05
rs12712997	nothing <0.01 within 10k	2	48085061	intron of FBXO11	Rye et al 2012	
rs1800587	nothing <0.01 within 10k	2	113542960	5' noncoding of IL1A	Joki-Erkkilä et al 2002	p<0.05
rs1143634	in gwas, p = 0.05	2	113590390	IL1Beta	McCormic et al 2011	
rs16944	nothing <0.01 within 10k	2	113594867	IL1Beta	Nokso-Koivisto et al 2014	
rs10497394	in gwas, p = 0.53	2	174297659	Intergenic	Allen et al, 2013	p=1.52x10-8
rs2276631	nothing <0.01 within 10k	2	219249013	SLC11A1	Rye et al 2013	
rs3731865	nothing <0.01 within 10k	2	219250003	SLC11A1	Rye et al 2013	
rs2695343	nothing <0.01 within 10k	2	219255423	SLC11A1	Rye et al 2013	
rs34448891	nothing <0.01 within 10k	2	219246649-219246650	SLC11A1	Rye et al 2013	
rs3732378	in gwas, p = 0.45	3	39307162	CX3CR1	Nokso-Koivisto et al 2014	
rs6438779	in gwas, p = 0.49	3	122898729		Allen et al, 2013	p=2.9×10ī 6
rs11097383	nothing <0.01 within 10k	4	94583840	GRID2	Rye et al 2012	p=5.97x10-5
rs10008015	nothing <0.01 within 10k	4	106005247	Intergenic	Rye et al 2012	p=3.89x10-5
rs1859161	in gwas, p = 0.46	4	106042692	Intergenic	Rye et al 2012	p=1.90x10-5
rs11940126	in gwas, p = 0.97	4	186790909	SORBS2	Rye et al 2012	p=5.70x10-5
rs386057	in gwas, p = 0.62	5	685748	TPPP	Allen et al, 2013	p=6.94x10-5
rs1800750	nothing <0.01 within 10k	5	31542963	promoter of TNFa	Emonts et al 2007b	
rs2569190	nothing <0.01 within 10k	5	140012916	5' noncoding of CD14	Wiertsema et al 2006	p<0.05
rs1800629	nothing <0.01 within 10k	6	31543031	promoter of TNFa	Patel et al 2006	
rs361525	nothing <0.01 within 10k	6	31543101	promoter of TNFa	Emonts et al 2007b	
rs10499006	in gwas, p = 0.11	6	96293482		Allen et al, 2013	p=2.1x10ī 6
rs1800795	nothing <0.01 within 10k	7	22766645	promoter of IL6	Emonts et al 2007b	p<0.05
rs10242197	nothing <0.01 within 10k	7	90259912	CDK14	Rye et al 2012	p=4.38x10-5
rs10488001	in gwas, p = 0.34	7	90656040	CDK14	Rye et al 2012	p=4.53x10-5
rs1799889	nothing <0.01 within 10k	7	100769711	promoter of SERPINE1	Emonts et al 2007	p<0.05
rs1496306	nothing <0.01 within 10k	8	50540708	Intergenic	Rye et al 2012	p=1.63x10-5
rs2132528	nothing <0.01 within 10k	8	50605655	Intergenic	Rye et al 2012	p=2.41x10-5
rs13438948	nothing <0.01 within 10k	8	87858778	Intergenic	Rye et al 2012	p=1.19x10-5
rs7846284	in gwas, p = 0.15	8	130370235	Intergenic	Rye et al 2012	p=4.06x10-5
rs7846684	in gwas, p = 0.14	8	130370414	Intergenic	Rye et al 2012	p=3.60x10-5
rs11790808	in gwas, p = 0.40	9	94588498	ROR2	Rye et al 2012	p=1.15x10-5
rs11536857	nothing <0.01 within 10k	9	120464136	promoter of TLR4		
rs12377632	nothing <0.01 within 10k	9	120472730	intron of TLR4	Sale et al 2008	
rs2770146	nothing <0.01 within 10k	9	120473338	intron of TLR4	Sale et al 2008	
rs5030717	nothing <0.01 within 10k	9	120473834	intron of TLR4	Sale et al 2008	
rs4986790	nothing <0.01 within 10k	9	120475302	exon of TLR4	Emonts et al 2007b	
rs10776851	nothing <0.01 within 10k	9	138718121	CAMSAP1	Rye et al 2012	p=2.13x10-5
rs4575213	in gwas, p = 0.76	10	53425328	PRKG1	Rye et al 2012	p=4.65x10-5
rs930507	nothing <0.01 within 10k	10	54528266	exon of MBL2	Wiertsema et al 2006c	
rs1800451	nothing <0.01 within 10k	10	54531226	exon of MBL2	Wiertsema et al 2006c	
rs1800450	in gwas, p = 0.32	10	54531235	exon of MBL2	Wiertsema et al 2006c	

rs5030737	nothing <0.01 within 10k	10	54531242	**exon of MBL2**	Wiertsema et al 2006c	
rs7096206	nothing <0.01 within 10k	10	54531685	**promoter of MBL2**	Wiertsema et al 2006c	
rs11003125	nothing <0.01 within 10k	10	54532014	**promoter of MBL2**	Wiertsema et al 2006c	
rs1965708	nothing <0.01 within 10k	10	81317045	**exon of SFTPA2**	Pettigrew et al 2007	p<0.05
rs1965707	nothing <0.01 within 10k	10	81317292	**exon of SFTPA2**	Pettigrew et al 2007	p<0.05
rs1059046	nothing <0.01 within 10k	10	81319214	**exon of SFTPA2**	Pettigrew et al 2007	p<0.05
rs1059047	nothing <0.01 within 10k	10	81371637	**exon of SFTPA1**	Pettigrew et al 2007	p<0.05
rs1136450	nothing <0.01 within 10k	10	81371729	**exon of SFTPA1**	Pettigrew et al 2007	p<0.05
rs1136451	nothing <0.01 within 10k	10	81372081	**exon of SFTPA1**	Pettigrew et al 2007	p<0.05
rs4253527	nothing <0.01 within 10k	10	81373777	**exon of SFTPA1**	Pettigrew et al 2007	p<0.05
rs1051246	nothing <0.01 within 10k	10	81697818	**SFTPD**	Sale MM et al 2011	
rs10884043	in gwas, p = 0.81	10	106531703	**SORCS3**	Rye et al 2012	p=2.74x10-5
rs4556466	in gwas, p = 0.44	10	106558025	**SORCS3**	Rye et al 2012	p=2.47x10-5
rs1059057	nothing <0.01 within 10k	10	N/A	**exon of SFTPA1**	Pettigrew et al 2007	p<0.05
rs7396030	in gwas, p = 0.82	11	1083364	**MUC2**	Sale MM et al 2011	p<0.05
rs4963049	nothing <0.01 within 10k	11	1236427	**MUC5AC/B**	MacArthur et al 2014	
rs2075859	in gwas, p = 0.56	11	1250488	**MUC5AC,MUC5**	Sale MM et al 2011	
rs2735733	nothing <0.01 within 10k	11	1261640	**MUC5B**	Sale et al 2008	p<0.05
rs2430561	nothing <0.01 within 10k	12	68552522	**intron of IFNG**	Gentile et al 2003	
rs9564897	nothing <0.01 within 10k	13	72912338	**Intergenic**	Rye et al 2012	p=1.98x10-5
rs10775247	in gwas, p = 0.61	15	90126121	**TICRR**	Allen et al, 2013	p=3X10-4
rs1110060	in gwas, p = 0.66	15	90190048	**KIF7**	Allen et al, 2013	p=4.09x10ī 5
rs1792658	in gwas, p = 0.24	18	45382605	**intron of SMAD2**	Rye et al 2013	p<0.05
rs8097137	rs1942159, rs3923814 and rs11660291 p<0.01	18	45483890	**SMAD2**	MacArthur et al 2014	
rs10502913	nothing <0.01 within 10k	18	48568271	**intron of SMAD4**	Rye et al 2014	p<0.05
rs17663887	nothing <0.01 within 10k	18	48589718	**SMAD4**	MacArthur et al 2014	
rs8100085	nothing <0.01 within 10k	19	35523119	**SCN1B**	Sale MM et al 2011	
rs1800471	nothing <0.01 within 10k	19	41858876	**exon of TGFb**	Gentile et al 2003	p<0.05
rs1982073	nothing <0.01 within 10k	19	41858921	**exon of TGFb**	Patel et al 2006	
rs17396317	nothing <0.01 within 10k	20	31790377	**BPIFA4Pc**	Rye et al 2012	p=5.15x10-5
rs1894516	nothing <0.01 within 10k	X	124133621	**Intergenic**	Rye et al 2012	p=6.64x10-6

Supplementary figure 1

A

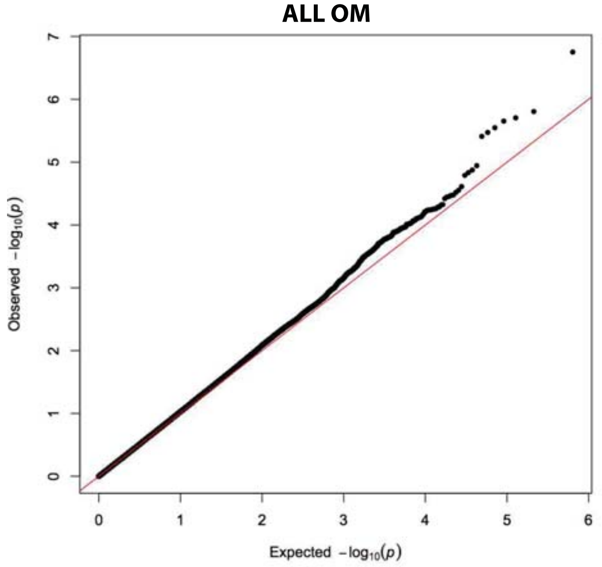

B

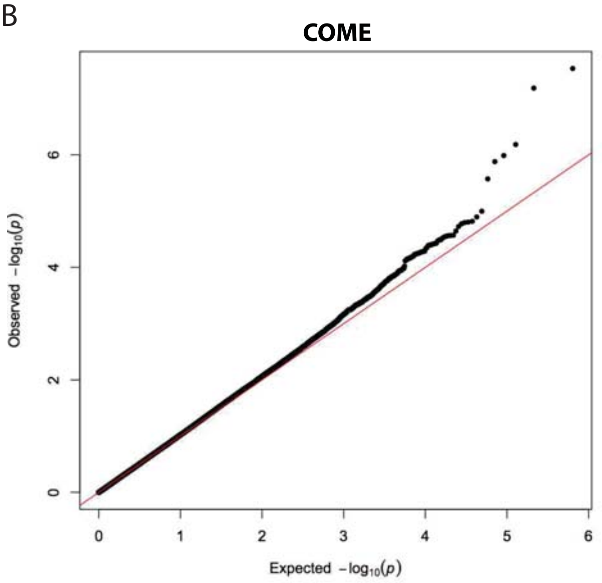

C

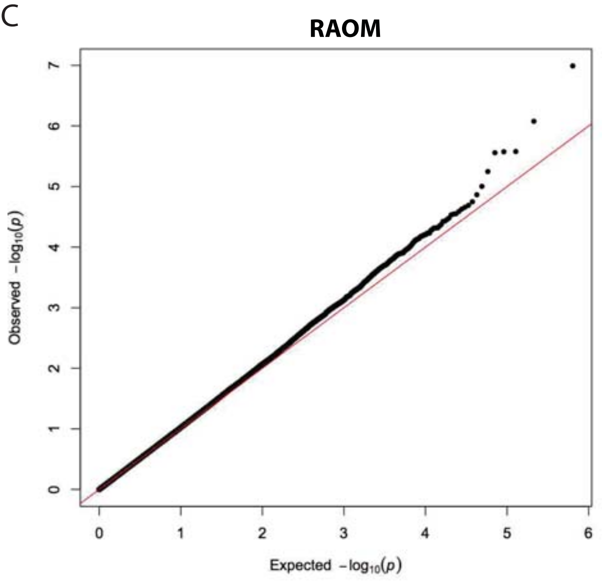

Supplementary figure 2

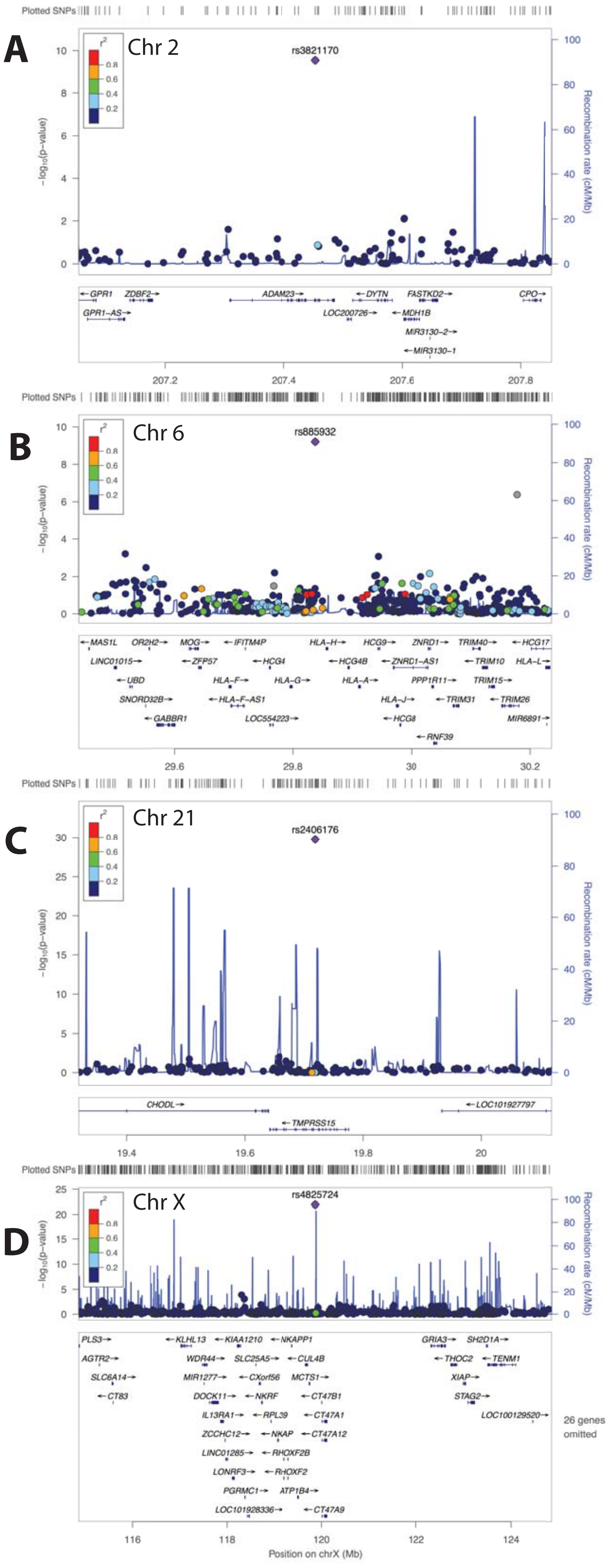

Supplementary figure 3

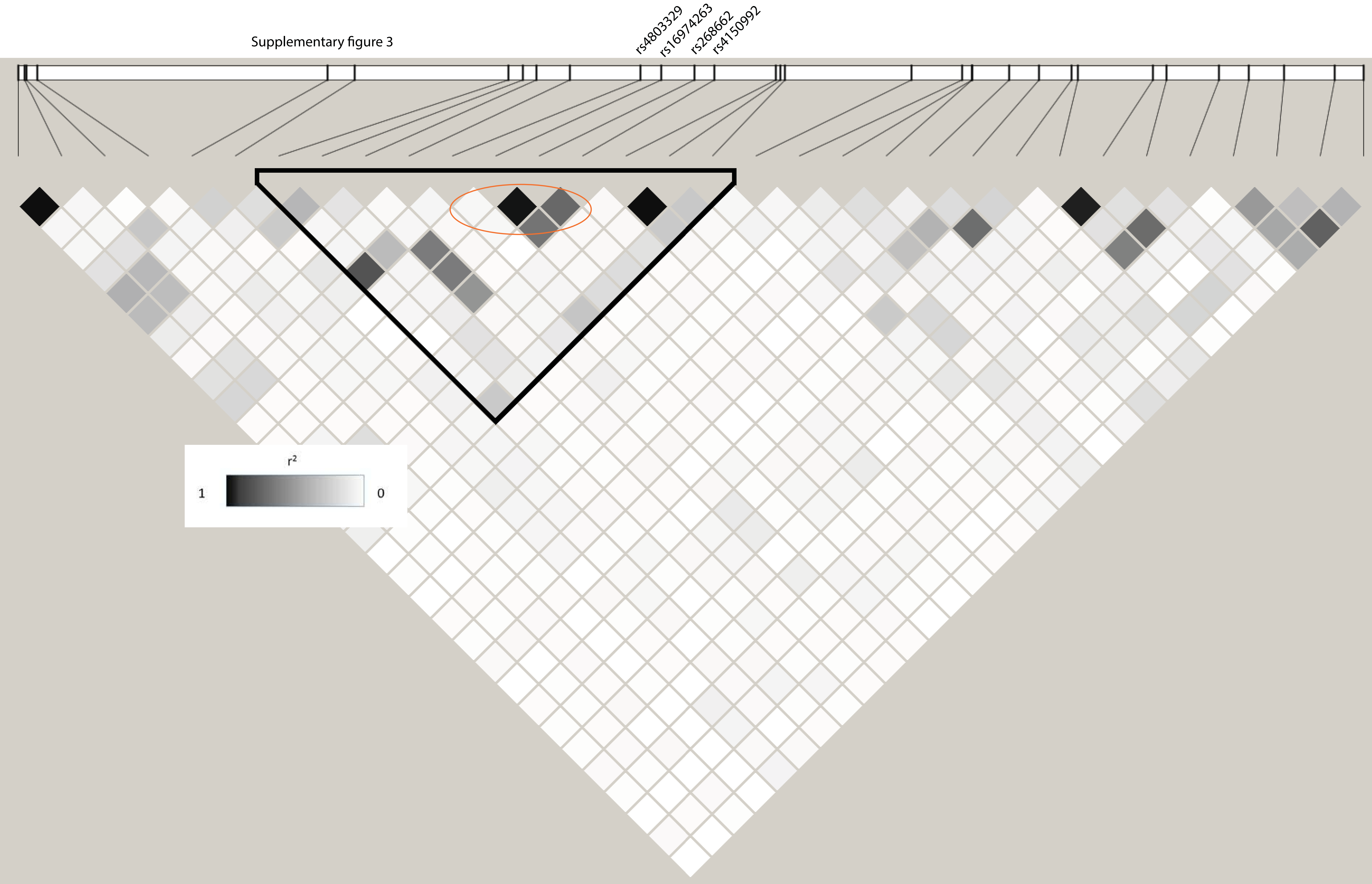

Supplementary figure 4

GO ANALYSIS OF KNOWN OTITIS MEDIA GENES

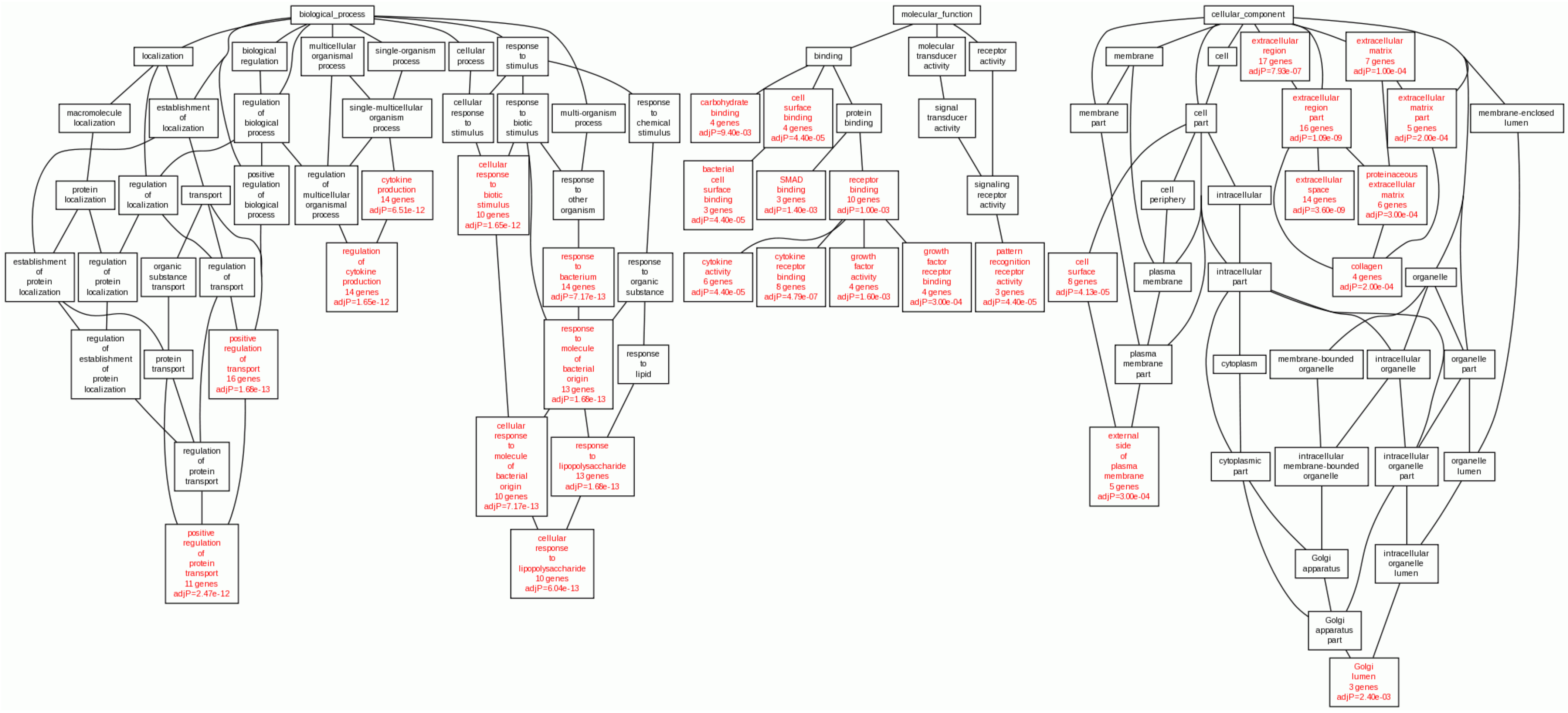

Database:biological process Name:response to lipopolysaccharide ID:GO:0032496
C=212; O=13; E=0.46; R=28.04; rawP=2.34e-16; adjP=1.68e-13

Index	UserID	Value	Gene Symbol	Gene Name	EntrezGen	Ensembl
1	TGFB1	NA	TGFB1	transforming growth factor, beta 1	7040	ENSG00000105329
2	IFNG	NA	IFNG	interferon, gamma	3458	ENSG00000111537
3	TNF	NA	TNF	tumor necrosis factor	7124	ENSG00000232810
4	TLR2	NA	TLR2	toll-like receptor 2	7097	NULL
5	SLC11A1	NA	SLC11A1	solute carrier family 11 (proton-coupled divalent metal ion transporters), member 1	6556	ENSG00000018280
6	CX3CR1	NA	CX3CR1	chemokine (C-X3-C motif) receptor 1	1524	ENSG00000168329
7	IL6	NA	IL6	interleukin 6 (interferon, beta 2)	3569	ENSG00000136244
8	SERPINE1	NA	SERPINE1	serpin peptidase inhibitor, clade E (nexin, plasminogen activator inhibitor type 1), member 1	5054	ENSG00000106366
9	IL10	NA	IL10	interleukin 10	3586	ENSG00000136634
10	CD14	NA	CD14	CD14 molecule	929	ENSG00000170458
11	IL1A	NA	IL1A	interleukin 1, alpha	3552	ENSG00000115008
12	IL1B	NA	IL1B	interleukin 1, beta	3553	ENSG00000125538
13	TLR4	NA	TLR4	toll-like receptor 4	7099	ENSG00000136869

Database:biological process Name:positive regulation of transport ID:GO:0051050
C=482; O=16; E=1.05; R=15.18; rawP=5.58e-16; adjP=1.68e-13

Index	UserID	Value	Gene Symbol	Gene Name	EntrezGen	Ensembl
1	TGFB1	NA	TGFB1	transforming growth factor, beta 1	7040	ENSG00000105329
2	IFNG	NA	IFNG	interferon, gamma	3458	ENSG00000111537
3	TNF	NA	TNF	tumor necrosis factor	7124	ENSG00000232810
4	MBL2	NA	MBL2	mannose-binding lectin (protein C) 2, soluble	4153	ENSG00000165471
5	TLR2	NA	TLR2	toll-like receptor 2	7097	NULL
6	SLC11A1	NA	SLC11A1	solute carrier family 11 (proton-coupled divalent metal ion transporters), member 1	6556	ENSG00000018280
7	SCN1B	NA	SCN1B	sodium channel, voltage-gated, type I, beta subunit	6324	ENSG00000105711
8	IL6	NA	IL6	interleukin 6 (interferon, beta 2)	3569	ENSG00000136244
9	SERPINE1	NA	SERPINE1	serpin peptidase inhibitor, clade E (nexin, plasminogen activator inhibitor type 1), member 1	5054	ENSG00000106366
10	IL10	NA	IL10	interleukin 10	3586	ENSG00000136634
11	CD14	NA	CD14	CD14 molecule	929	ENSG00000170458
12	SFTPD	NA	SFTPD	surfactant protein D	6441	ENSG00000133661
13	IL1A	NA	IL1A	interleukin 1, alpha	3552	ENSG00000115008
14	IL1B	NA	IL1B	interleukin 1, beta	3553	ENSG00000125538
15	TLR4	NA	TLR4	toll-like receptor 4	7099	ENSG00000136869
16	SMAD4	NA	SMAD4	SMAD family member 4	4089	ENSG00000141646

Database:biological process Name:response to molecule of bacterial origin ID:GO:0002237
C=225; O=13; E=0.49; R=26.42; rawP=5.11e-16; adjP=1.68e-13

Index	UserID	Value	Gene Symbol	Gene Name	EntrezGen	Ensembl
1	TGFB1	NA	TGFB1	transforming growth factor, beta 1	7040	ENSG00000105329
2	IFNG	NA	IFNG	interferon, gamma	3458	ENSG00000111537
3	TNF	NA	TNF	tumor necrosis factor	7124	ENSG00000232810
4	TLR2	NA	TLR2	toll-like receptor 2	7097	NULL
5	SLC11A1	NA	SLC11A1	solute carrier family 11 (proton-coupled divalent metal ion transporters), member 1	6556	ENSG00000018280
6	CX3CR1	NA	CX3CR1	chemokine (C-X3-C motif) receptor 1	1524	ENSG00000168329
7	IL6	NA	IL6	interleukin 6 (interferon, beta 2)	3569	ENSG00000136244
8	SERPINE1	NA	SERPINE1	serpin peptidase inhibitor, clade E (nexin, plasminogen activator inhibitor type 1), member 1	5054	ENSG00000106366
9	IL10	NA	IL10	interleukin 10	3586	ENSG00000136634
10	CD14	NA	CD14	CD14 molecule	929	ENSG00000170458
11	IL1A	NA	IL1A	interleukin 1, alpha	3552	ENSG00000115008
12	IL1B	NA	IL1B	interleukin 1, beta	3553	ENSG00000125538
13	TLR4	NA	TLR4	toll-like receptor 4	7099	ENSG00000136869

Database:biological process Name:cellular response to lipopolysaccharide ID:GO:0071222
C=90; O=10; E=0.20; R=50.80; rawP=2.68e-15; adjP=6.04e-13

Index	UserID	Value	Gene Symbol	Gene Name	EntrezGen	Ensembl
1	TGFB1	NA	TGFB1	transforming growth factor, beta 1	7040	ENSG00000105329
2	IFNG	NA	IFNG	interferon, gamma	3458	ENSG00000111537
3	TNF	NA	TNF	tumor necrosis factor	7124	ENSG00000232810
4	TLR2	NA	TLR2	toll-like receptor 2	7097	NULL
5	CX3CR1	NA	CX3CR1	chemokine (C-X3-C motif) receptor 1	1524	ENSG00000168329
6	SERPINE1	NA	SERPINE1	serpin peptidase inhibitor, clade E (nexin, plasminogen activator inhibitor type 1), member 1	5054	ENSG00000106366
7	CD14	NA	CD14	CD14 molecule	929	ENSG00000170458
8	IL10	NA	IL10	interleukin 10	3586	ENSG00000136634
9	IL1B	NA	IL1B	interleukin 1, beta	3553	ENSG00000125538
10	TLR4	NA	TLR4	toll-like receptor 4	7099	ENSG00000136869

Database:biological process Name:cellular response to molecule of bacterial origin ID:GO:0071219
C=95; O=10; E=0.21; R=48.12; rawP=4.70e-15; adjP=7.17e-13

Index	UserID	Value	Gene Symbol	Gene Name	EntrezGen	Ensembl
1	TGFB1	NA	TGFB1	transforming growth factor, beta 1	7040	ENSG00000105329
2	IFNG	NA	IFNG	interferon, gamma	3458	ENSG00000111537
3	TNF	NA	TNF	tumor necrosis factor	7124	ENSG00000232810
4	TLR2	NA	TLR2	toll-like receptor 2	7097	NULL
5	CX3CR1	NA	CX3CR1	chemokine (C-X3-C motif) receptor 1	1524	ENSG00000168329
6	SERPINE1	NA	SERPINE1	serpin peptidase inhibitor, clade E (nexin, plasminogen activator inhibitor type 1), member 1	5054	ENSG00000106366
7	CD14	NA	CD14	CD14 molecule	929	ENSG00000170458
8	IL10	NA	IL10	interleukin 10	3586	ENSG00000136634
9	IL1B	NA	IL1B	interleukin 1, beta	3553	ENSG00000125538
10	TLR4	NA	TLR4	toll-like receptor 4	7099	ENSG00000136869

Database:biological process Name:response to bacterium ID:GO:0009617
C=349; O=14; E=0.76; R=18.34; rawP=4.77e-15; adjP=7.17e-13

Index	UserID	Value	Gene Symbol	Gene Name	EntrezGen	Ensembl
1	TGFB1	NA	TGFB1	transforming growth factor, beta 1	7040	ENSG00000105329
2	IFNG	NA	IFNG	interferon, gamma	3458	ENSG00000111537
3	TNF	NA	TNF	tumor necrosis factor	7124	ENSG00000232810
4	MBL2	NA	MBL2	mannose-binding lectin (protein C) 2, soluble	4153	ENSG00000165471
5	TLR2	NA	TLR2	toll-like receptor 2	7097	NULL
6	SLC11A1	NA	SLC11A1	solute carrier family 11 (proton-coupled divalent metal ion transporters), member 1	6556	ENSG00000018280
7	CX3CR1	NA	CX3CR1	chemokine (C-X3-C motif) receptor 1	1524	ENSG00000168329
8	IL6	NA	IL6	interleukin 6 (interferon, beta 2)	3569	ENSG00000136244
9	SERPINE1	NA	SERPINE1	serpin peptidase inhibitor, clade E (nexin, plasminogen activator inhibitor type 1), member 1	5054	ENSG00000106366
10	IL10	NA	IL10	interleukin 10	3586	ENSG00000136634
11	CD14	NA	CD14	CD14 molecule	929	ENSG00000170458
12	IL1A	NA	IL1A	interleukin 1, alpha	3552	ENSG00000115008
13	IL1B	NA	IL1B	interleukin 1, beta	3553	ENSG00000125538
14	TLR4	NA	TLR4	toll-like receptor 4	7099	ENSG00000136869

Database:biological process Name:cellular response to biotic stimulus ID:GO:0071216
C=106; O=10; E=0.23; R=43.13; rawP=1.46e-14; adjP=1.65e-12

Index	UserID	Value	Gene Symbol	Gene Name	EntrezGen	Ensembl

1	TGFB1	NA	TGFB1	transforming growth factor, beta 1	7040	ENSG00000105329
2	IFNG	NA	IFNG	interferon, gamma	3458	ENSG00000111537
3	TNF	NA	TNF	tumor necrosis factor	7124	ENSG00000232810
4	TLR2	NA	TLR2	toll-like receptor 2	7097	NULL
5	CX3CR1	NA	CX3CR1	chemokine (C-X3-C motif) receptor 1	1524	ENSG00000168329
6	SERPINE1	NA	SERPINE1	serpin peptidase inhibitor, clade E (nexin, plasminogen activator inhibitor type 1), member 1	5054	ENSG00000106366
7	CD14	NA	CD14	CD14 molecule	929	ENSG00000170458
8	IL10	NA	IL10	interleukin 10	3586	ENSG00000136634
9	IL1B	NA	IL1B	interleukin 1, beta	3553	ENSG00000125538
10	TLR4	NA	TLR4	toll-like receptor 4	7099	ENSG00000136869

Database:biological process Name:regulation of cytokine production ID:GO:0001817

C=378; O=14; E=0.83; R=16.93; rawP=1.44e-14; adjP=1.65e-12

Index	UserID	Value	Gene Symbol	Gene Name	EntrezGen	Ensembl
1	TGFB1	NA	TGFB1	transforming growth factor, beta 1	7040	ENSG00000105329
2	IFNG	NA	IFNG	interferon, gamma	3458	ENSG00000111537
3	TNF	NA	TNF	tumor necrosis factor	7124	ENSG00000232810
4	TLR2	NA	TLR2	toll-like receptor 2	7097	NULL
5	SLC11A1	NA	SLC11A1	solute carrier family 11 (proton-coupled divalent metal ion transporters), member 1	6556	ENSG00000018280
6	IL6	NA	IL6	interleukin 6 (interferon, beta 2)	3569	ENSG00000136244
7	SERPINE1	NA	SERPINE1	serpin peptidase inhibitor, clade E (nexin, plasminogen activator inhibitor type 1), member 1	5054	ENSG00000106366
8	IL10	NA	IL10	interleukin 10	3586	ENSG00000136634
9	CD14	NA	CD14	CD14 molecule	929	ENSG00000170458
10	SFTPD	NA	SFTPD	surfactant protein D	6441	ENSG00000133661
11	IL1A	NA	IL1A	interleukin 1, alpha	3552	ENSG00000115008
12	IL1B	NA	IL1B	interleukin 1, beta	3553	ENSG00000125538
13	TLR4	NA	TLR4	toll-like receptor 4	7099	ENSG00000136869
14	SMAD4	NA	SMAD4	SMAD family member 4	4089	ENSG00000141646

Database:biological process Name:positive regulation of protein transport ID:GO:0051222

C=163; O=11; E=0.36; R=30.85; rawP=2.46e-14; adjP=2.47e-12

Index	UserID	Value	Gene Symbol	Gene Name	EntrezGen	Ensembl
1	TGFB1	NA	TGFB1	transforming growth factor, beta 1	7040	ENSG00000105329
2	IFNG	NA	IFNG	interferon, gamma	3458	ENSG00000111537
3	TNF	NA	TNF	tumor necrosis factor	7124	ENSG00000232810
4	TLR2	NA	TLR2	toll-like receptor 2	7097	NULL
5	IL6	NA	IL6	interleukin 6 (interferon, beta 2)	3569	ENSG00000136244
6	CD14	NA	CD14	CD14 molecule	929	ENSG00000170458
7	IL10	NA	IL10	interleukin 10	3586	ENSG00000136634
8	IL1A	NA	IL1A	interleukin 1, alpha	3552	ENSG00000115008
9	IL1B	NA	IL1B	interleukin 1, beta	3553	ENSG00000125538
10	TLR4	NA	TLR4	toll-like receptor 4	7099	ENSG00000136869
11	SMAD4	NA	SMAD4	SMAD family member 4	4089	ENSG00000141646

Database:biological process Name:cytokine production ID:GO:0001816

C=425; O=14; E=0.93; R=15.06; rawP=7.22e-14; adjP=6.51e-12

Index	UserID	Value	Gene Symbol	Gene Name	EntrezGen	Ensembl
1	TGFB1	NA	TGFB1	transforming growth factor, beta 1	7040	ENSG00000105329
2	IFNG	NA	IFNG	interferon, gamma	3458	ENSG00000111537
3	TNF	NA	TNF	tumor necrosis factor	7124	ENSG00000232810
4	TLR2	NA	TLR2	toll-like receptor 2	7097	NULL
5	SLC11A1	NA	SLC11A1	solute carrier family 11 (proton-coupled divalent metal ion transporters), member 1	6556	ENSG00000018280
6	IL6	NA	IL6	interleukin 6 (interferon, beta 2)	3569	ENSG00000136244
7	SERPINE1	NA	SERPINE1	serpin peptidase inhibitor, clade E (nexin, plasminogen activator inhibitor type 1), member 1	5054	ENSG00000106366
8	IL10	NA	IL10	interleukin 10	3586	ENSG00000136634
9	CD14	NA	CD14	CD14 molecule	929	ENSG00000170458
10	SFTPD	NA	SFTPD	surfactant protein D	6441	ENSG00000133661
11	IL1A	NA	IL1A	interleukin 1, alpha	3552	ENSG00000115008
12	IL1B	NA	IL1B	interleukin 1, beta	3553	ENSG00000125538
13	TLR4	NA	TLR4	toll-like receptor 4	7099	ENSG00000136869
14	SMAD4	NA	SMAD4	SMAD family member 4	4089	ENSG00000141646

Database:molecular function Name:cytokine receptor binding ID:GO:0005126

C=214; O=8; E=0.46; R=17.31; rawP=1.33e-08; adjP=4.79e-07

Index	UserID	Value	Gene Symbol	Gene Name	EntrezGen	Ensembl
1	TGFB1	NA	TGFB1	transforming growth factor, beta 1	7040	ENSG00000105329
2	IFNG	NA	IFNG	interferon, gamma	3458	ENSG00000111537
3	TNF	NA	TNF	tumor necrosis factor	7124	ENSG00000232810
4	SMAD2	NA	SMAD2	SMAD family member 2	4087	ENSG00000175387
5	IL10	NA	IL10	interleukin 10	3586	ENSG00000136634
6	IL1A	NA	IL1A	interleukin 1, alpha	3552	ENSG00000115008
7	IL1B	NA	IL1B	interleukin 1, beta	3553	ENSG00000125538
8	IL6	NA	IL6	interleukin 6 (interferon, beta 2)	3569	ENSG00000136244

Database:molecular function Name:cytokine activity ID:GO:0005125

C=207; O=6; E=0.45; R=13.42; rawP=4.69e-06; adjP=4.40e-05

Index	UserID	Value	Gene Symbol	Gene Name	EntrezGen	Ensembl
1	IFNG	NA	IFNG	interferon, gamma	3458	ENSG00000111537
2	TNF	NA	TNF	tumor necrosis factor	7124	ENSG00000232810
3	IL10	NA	IL10	interleukin 10	3586	ENSG00000136634
4	IL1A	NA	IL1A	interleukin 1, alpha	3552	ENSG00000115008
5	IL1B	NA	IL1B	interleukin 1, beta	3553	ENSG00000125538
6	IL6	NA	IL6	interleukin 6 (interferon, beta 2)	3569	ENSG00000136244

Database:molecular function Name:cell surface binding ID:GO:0043498

C=54; O=4; E=0.12; R=34.31; rawP=5.28e-06; adjP=4.40e-05

Index	UserID	Value	Gene Symbol	Gene Name	EntrezGen	Ensembl
1	TGFB1	NA	TGFB1	transforming growth factor, beta 1	7040	ENSG00000105329
2	MBL2	NA	MBL2	mannose-binding lectin (protein C) 2, soluble	4153	ENSG00000165471
3	TLR2	NA	TLR2	toll-like receptor 2	7097	NULL
4	SFTPD	NA	SFTPD	surfactant protein D	6441	ENSG00000133661

Database:molecular function Name:bacterial cell surface binding ID:GO:0051635

C=17; O=3; E=0.04; R=81.73; rawP=6.11e-06; adjP=4.40e-05

Index	UserID	Value	Gene Symbol	Gene Name	EntrezGen	Ensembl
1	MBL2	NA	MBL2	mannose-binding lectin (protein C) 2, soluble	4153	ENSG00000165471
2	TLR2	NA	TLR2	toll-like receptor 2	7097	NULL
3	SFTPD	NA	SFTPD	surfactant protein D	6441	ENSG00000133661

Database:molecular function Name:pattern recognition receptor activity ID:GO:0008329

C=15; O=3; E=0.03; R=92.63; rawP=4.10e-06; adjP=4.40e-05

Index	UserID	Value	Gene Symbol	Gene Name	EntrezGen	Ensembl

1	TLR2	NA	TLR2	toll-like receptor 2	7097	NULL
2	CD14	NA	CD14	CD14 molecule	929	ENSG00000170458
3	TLR4	NA	TLR4	toll-like receptor 4	7099	ENSG00000136869

Database:molecular function Name:growth factor receptor binding ID:GO:0070851
C=98; O=4; E=0.21; R=18.90; rawP=5.64e-05; adjP=0.0003

Index	UserID	Value	Gene Symbol	Gene Name	EntrezGen	Ensembl
1	IL10	NA	IL10	interleukin 10	3586	ENSG00000136634
2	IL1A	NA	IL1A	interleukin 1, alpha	3552	ENSG00000115008
3	IL1B	NA	IL1B	interleukin 1, beta	3553	ENSG00000125538
4	IL6	NA	IL6	interleukin 6 (interferon, beta 2)	3569	ENSG00000136244

Database:molecular function Name:receptor binding ID:GO:0005102
C=1211; O=10; E=2.61; R=3.82; rawP=0.0002; adjP=0.0010

Index	UserID	Value	Gene Symbol	Gene Name	EntrezGen	Ensembl
1	TGFB1	NA	TGFB1	transforming growth factor, beta 1	7040	ENSG00000105329
2	IFNG	NA	IFNG	interferon, gamma	3458	ENSG00000111537
3	TNF	NA	TNF	tumor necrosis factor	7124	ENSG00000232810
4	MBL2	NA	MBL2	mannose-binding lectin (protein C) 2, soluble	4153	ENSG00000165471
5	SMAD2	NA	SMAD2	SMAD family member 2	4087	ENSG00000175387
6	IL6	NA	IL6	interleukin 6 (interferon, beta 2)	3569	ENSG00000136244
7	SERPINE1	NA	SERPINE1	serpin peptidase inhibitor, clade E (nexin, plasminogen activator inhibitor type 1), member 1	5054	ENSG00000106366
8	IL10	NA	IL10	interleukin 10	3586	ENSG00000136634
9	IL1A	NA	IL1A	interleukin 1, alpha	3552	ENSG00000115008
10	IL1B	NA	IL1B	interleukin 1, beta	3553	ENSG00000125538

Database:molecular function Name:SMAD binding ID:GO:0046332
C=63; O=3; E=0.14; R=22.05; rawP=0.0003; adjP=0.0014

Index	UserID	Value	Gene Symbol	Gene Name	EntrezGen	Ensembl
1	TGIF1	NA	TGIF1	TGFB-induced factor homeobox 1	7050	ENSG00000177426
2	SMAD2	NA	SMAD2	SMAD family member 2	4087	ENSG00000175387
3	SMAD4	NA	SMAD4	SMAD family member 4	4089	ENSG00000141646

Database:molecular function Name:growth factor activity ID:GO:0008083
C=164; O=4; E=0.35; R=11.30; rawP=0.0004; adjP=0.0016

Index	UserID	Value	Gene Symbol	Gene Name	EntrezGen	Ensembl
1	TGFB1	NA	TGFB1	transforming growth factor, beta 1	7040	ENSG00000105329
2	IL10	NA	IL10	interleukin 10	3586	ENSG00000136634
3	IL1B	NA	IL1B	interleukin 1, beta	3553	ENSG00000125538
4	IL6	NA	IL6	interleukin 6 (interferon, beta 2)	3569	ENSG00000136244

Database:molecular function Name:carbohydrate binding ID:GO:0030246
C=269; O=4; E=0.58; R=6.89; rawP=0.0026; adjP=0.0094

Index	UserID	Value	Gene Symbol	Gene Name	EntrezGen	Ensembl
1	MBL2	NA	MBL2	mannose-binding lectin (protein C) 2, soluble	4153	ENSG00000165471
2	SFTPA1	NA	SFTPA1	surfactant protein A1	653509	ENSG00000122852
3	SFTPD	NA	SFTPD	surfactant protein D	6441	ENSG00000133661
4	SFTPA2	NA	SFTPA2	surfactant protein A2	729238	ENSG00000185303

Database:cellular component Name:extracellular region part ID:GO:0044421
C=1099; O=16; E=2.06; R=7.76; rawP=1.79e-11; adjP=1.09e-09

Index	UserID	Value	Gene Symbol	Gene Name	EntrezGen	Ensembl
1	TGFB1	NA	TGFB1	transforming growth factor, beta 1	7040	ENSG00000105329
2	IFNG	NA	IFNG	interferon, gamma	3458	ENSG00000111537
3	TNF	NA	TNF	tumor necrosis factor	7124	ENSG00000232810
4	A2ML1	NA	A2ML1	alpha-2-macroglobulin-like 1	144568	ENSG00000166535
5	SFTPA1	NA	SFTPA1	surfactant protein A1	653509	ENSG00000122852
6	SFTPA2	NA	SFTPA2	surfactant protein A2	729238	ENSG00000185303
7	MUC2	NA	MUC2	mucin 2, oligomeric mucus/gel-forming	4583	ENSG00000198788
8	IL10	NA	IL10	interleukin 10	3586	ENSG00000136634
9	CD14	NA	CD14	CD14 molecule	929	ENSG00000170458
10	SFTPD	NA	SFTPD	surfactant protein D	6441	ENSG00000133661
11	IL1A	NA	IL1A	interleukin 1, alpha	3552	ENSG00000115008
12	IL1B	NA	IL1B	interleukin 1, beta	3553	ENSG00000125538
13	MUC5AC	NA	MUC5AC	mucin 5AC, oligomeric mucus/gel-forming	4586	NULL
14	MBL2	NA	MBL2	mannose-binding lectin (protein C) 2, soluble	4153	ENSG00000165471
15	IL6	NA	IL6	interleukin 6 (interferon, beta 2)	3569	ENSG00000136244
16	SERPINE1	NA	SERPINE1	serpin peptidase inhibitor, clade E (nexin, plasminogen activator inhibitor type 1), member 1	5054	ENSG00000106366

Database:cellular component Name:extracellular space ID:GO:0005615
C=856; O=14; E=1.61; R=8.72; rawP=1.18e-10; adjP=3.60e-09

Index	UserID	Value	Gene Symbol	Gene Name	EntrezGen	Ensembl
1	TGFB1	NA	TGFB1	transforming growth factor, beta 1	7040	ENSG00000105329
2	IFNG	NA	IFNG	interferon, gamma	3458	ENSG00000111537
3	TNF	NA	TNF	tumor necrosis factor	7124	ENSG00000232810
4	MBL2	NA	MBL2	mannose-binding lectin (protein C) 2, soluble	4153	ENSG00000165471
5	A2ML1	NA	A2ML1	alpha-2-macroglobulin-like 1	144568	ENSG00000166535
6	SFTPA1	NA	SFTPA1	surfactant protein A1	653509	ENSG00000122852
7	SFTPA2	NA	SFTPA2	surfactant protein A2	729238	ENSG00000185303
8	IL6	NA	IL6	interleukin 6 (interferon, beta 2)	3569	ENSG00000136244
9	SERPINE1	NA	SERPINE1	serpin peptidase inhibitor, clade E (nexin, plasminogen activator inhibitor type 1), member 1	5054	ENSG00000106366
10	IL10	NA	IL10	interleukin 10	3586	ENSG00000136634
11	CD14	NA	CD14	CD14 molecule	929	ENSG00000170458
12	SFTPD	NA	SFTPD	surfactant protein D	6441	ENSG00000133661
13	IL1A	NA	IL1A	interleukin 1, alpha	3552	ENSG00000115008
14	IL1B	NA	IL1B	interleukin 1, beta	3553	ENSG00000125538

Database:cellular component Name:extracellular region ID:GO:0005576
C=2140; O=17; E=4.02; R=4.23; rawP=3.90e-08; adjP=7.93e-07

Index	UserID	Value	Gene Symbol	Gene Name	EntrezGen	Ensembl
1	TGFB1	NA	TGFB1	transforming growth factor, beta 1	7040	ENSG00000105329
2	IFNG	NA	IFNG	interferon, gamma	3458	ENSG00000111537
3	TNF	NA	TNF	tumor necrosis factor	7124	ENSG00000232810
4	A2ML1	NA	A2ML1	alpha-2-macroglobulin-like 1	144568	ENSG00000166535
5	SFTPA1	NA	SFTPA1	surfactant protein A1	653509	ENSG00000122852
6	SFTPA2	NA	SFTPA2	surfactant protein A2	729238	ENSG00000185303
7	MUC2	NA	MUC2	mucin 2, oligomeric mucus/gel-forming	4583	ENSG00000198788
8	IL10	NA	IL10	interleukin 10	3586	ENSG00000136634
9	CD14	NA	CD14	CD14 molecule	929	ENSG00000170458
10	SFTPD	NA	SFTPD	surfactant protein D	6441	ENSG00000133661
11	IL1A	NA	IL1A	interleukin 1, alpha	3552	ENSG00000115008
12	IL1B	NA	IL1B	interleukin 1, beta	3553	ENSG00000125538

13	MUC5AC	NA	MUC5AC	mucin 5AC, oligomeric mucus/gel-forming	4586	NULL
14	MBL2	NA	MBL2	mannose-binding lectin (protein C) 2, soluble	4153	ENSG00000165471
15	SCN1B	NA	SCN1B	sodium channel, voltage-gated, type I, beta subunit	6324	ENSG00000105711
16	IL6	NA	IL6	interleukin 6 (interferon, beta 2)	3569	ENSG00000136244
17	SERPINE1	NA	SERPINE1	serpin peptidase inhibitor, clade E (nexin, plasminogen activator inhibitor type 1), member 1	5054	ENSG00000106366

Database:cellular component Name:cell surface ID:GO:0009986

C=495; O=8; E=0.93; R=8.61; rawP=2.71e-06; adjP=4.13e-05

Index	UserID	Value	Gene Symbol	Gene Name	EntrezGen	Ensembl
1	TGFB1	NA	TGFB1	transforming growth factor, beta 1	7040	ENSG00000105329
2	IFNG	NA	IFNG	interferon, gamma	3458	ENSG00000111537
3	TNF	NA	TNF	tumor necrosis factor	7124	ENSG00000232810
4	TLR2	NA	TLR2	toll-like receptor 2	7097	NULL
5	CD14	NA	CD14	CD14 molecule	929	ENSG00000170458
6	IL1A	NA	IL1A	interleukin 1, alpha	3552	ENSG00000115008
7	TLR4	NA	TLR4	toll-like receptor 4	7099	ENSG00000136869
8	IL6	NA	IL6	interleukin 6 (interferon, beta 2)	3569	ENSG00000136244

Database:cellular component Name:extracellular matrix ID:GO:0031012

C=426; O=7; E=0.80; R=8.76; rawP=1.13e-05; adjP=0.0001

Index	UserID	Value	Gene Symbol	Gene Name	EntrezGen	Ensembl
1	TGFB1	NA	TGFB1	transforming growth factor, beta 1	7040	ENSG00000105329
2	MBL2	NA	MBL2	mannose-binding lectin (protein C) 2, soluble	4153	ENSG00000165471
3	SERPINE1	NA	SERPINE1	serpin peptidase inhibitor, clade E (nexin, plasminogen activator inhibitor type 1), member 1	5054	ENSG00000106366
4	SFTPA1	NA	SFTPA1	surfactant protein A1	653509	ENSG00000122852
5	SFTPD	NA	SFTPD	surfactant protein D	6441	ENSG00000133661
6	SFTPA2	NA	SFTPA2	surfactant protein A2	729238	ENSG00000185303
7	MUC5AC	NA	MUC5AC	mucin 5AC, oligomeric mucus/gel-forming	4586	NULL

Database:cellular component Name:extracellular matrix part ID:GO:0044420

C=185; O=5; E=0.35; R=14.40; rawP=2.26e-05; adjP=0.0002

Index	UserID	Value	Gene Symbol	Gene Name	EntrezGen	Ensembl
1	MBL2	NA	MBL2	mannose-binding lectin (protein C) 2, soluble	4153	ENSG00000165471
2	SFTPA1	NA	SFTPA1	surfactant protein A1	653509	ENSG00000122852
3	SFTPD	NA	SFTPD	surfactant protein D	6441	ENSG00000133661
4	SFTPA2	NA	SFTPA2	surfactant protein A2	729238	ENSG00000185303
5	MUC5AC	NA	MUC5AC	mucin 5AC, oligomeric mucus/gel-forming	4586	NULL

Database:cellular component Name:collagen ID:GO:0005581

C=89; O=4; E=0.17; R=23.95; rawP=2.23e-05; adjP=0.0002

Index	UserID	Value	Gene Symbol	Gene Name	EntrezGen	Ensembl
1	MBL2	NA	MBL2	mannose-binding lectin (protein C) 2, soluble	4153	ENSG00000165471
2	SFTPA1	NA	SFTPA1	surfactant protein A1	653509	ENSG00000122852
3	SFTPD	NA	SFTPD	surfactant protein D	6441	ENSG00000133661
4	SFTPA2	NA	SFTPA2	surfactant protein A2	729238	ENSG00000185303

Database:cellular component Name:external side of plasma membrane ID:GO:0009897

C=203; O=5; E=0.38; R=13.13; rawP=3.53e-05; adjP=0.0003

Index	UserID	Value	Gene Symbol	Gene Name	EntrezGen	Ensembl
1	IFNG	NA	IFNG	interferon, gamma	3458	ENSG00000111537
2	TNF	NA	TNF	tumor necrosis factor	7124	ENSG00000232810
3	TLR2	NA	TLR2	toll-like receptor 2	7097	NULL
4	TLR4	NA	TLR4	toll-like receptor 4	7099	ENSG00000136869
5	IL6	NA	IL6	interleukin 6 (interferon, beta 2)	3569	ENSG00000136244

Database:cellular component Name:proteinaceous extracellular matrix ID:GO:0005578

C=360; O=6; E=0.68; R=8.88; rawP=4.84e-05; adjP=0.0003

Index	UserID	Value	Gene Symbol	Gene Name	EntrezGen	Ensembl
1	TGFB1	NA	TGFB1	transforming growth factor, beta 1	7040	ENSG00000105329
2	MBL2	NA	MBL2	mannose-binding lectin (protein C) 2, soluble	4153	ENSG00000165471
3	SFTPA1	NA	SFTPA1	surfactant protein A1	653509	ENSG00000122852
4	SFTPD	NA	SFTPD	surfactant protein D	6441	ENSG00000133661
5	SFTPA2	NA	SFTPA2	surfactant protein A2	729238	ENSG00000185303
6	MUC5AC	NA	MUC5AC	mucin 5AC, oligomeric mucus/gel-forming	4586	NULL

Database:cellular component Name:Golgi lumen ID:GO:0005796

C=77; O=3; E=0.14; R=20.76; rawP=0.0004; adjP=0.0024

Index	UserID	Value	Gene Symbol	Gene Name	EntrezGen	Ensembl
1	TGFB1	NA	TGFB1	transforming growth factor, beta 1	7040	ENSG00000105329
2	MUC2	NA	MUC2	mucin 2, oligomeric mucus/gel-forming	4583	ENSG00000198788
3	MUC5AC	NA	MUC5AC	mucin 5AC, oligomeric mucus/gel-forming	4586	NULL
